# Techno-Economic Assessment of Industrial Symbiosis Between Steel and Urea Plants: The INITIATE Process

**DOI:** 10.1021/acs.energyfuels.5c04026

**Published:** 2025-11-12

**Authors:** Nicola Zecca, Leonie Lücking, H. A. J. van Dijk, Giampaolo Manzolini

**Affiliations:** † 119605 Politecnico di Milano, Dipartimento di Energia, via Lambruschini 4, Milano 20126, Italy; ‡ TNO, Westerduinweg 3, Petten 1755 LE, The Netherlands

## Abstract

The steelmaking and fertilizer industries accounted for approximately 10% of global anthropogenic CO_2_ emissions in 2024. This study examines an industrial symbiosis concept, termed INITIATE, which integrates these two sectors to enhance resource efficiency and to reduce CO_2_ emissions. The proposed system utilizes process gases from steel production as a feedstock for urea synthesis, using the sorption enhanced water gas shift (SEWGS) technology for simultaneous CO_2_ capture and production of a H_2_–N_2_ mixture. This stream is suitable for ammonia synthesis, which subsequently reacts with part of captured CO_2_ in a downstream urea production process. Two sizes of fertilizer production are analyzed: a small-scale configuration producing 224 t_urea_/day and a large-scale case with a production capacity of 1500 t_urea_/day. Simulation results indicate that the integrated symbiotic configuration of the INITIATE system enables substantial reductions in both the natural gas consumption and direct CO_2_ emissions. Under scenarios utilizing renewable electricity, the level of CO_2_ avoidance can reach up to 68%. The specific primary energy consumption per unit of CO_2_ avoided (SPECCA) ranges from −2.5 to 2.5 GJ/t_CO_2__. Negative values reflect a net reduction in primary energy demand, resulting from process integration and efficient resource utilization. From an economic perspective, the cost of CO_2_ avoidance is estimated at 24 €/t_CO_2__ for the small-scale plant, increasing to 130 €/t_CO_2__ for the large-scale configuration. Sensitivity analyses reveal that these costs are highly dependent on the prices of electricity and natural gas, with lower electricity prices and higher natural gas prices improving the economic performance of the INITIATE system compared with the base and reference cases.

## Graphical abstract

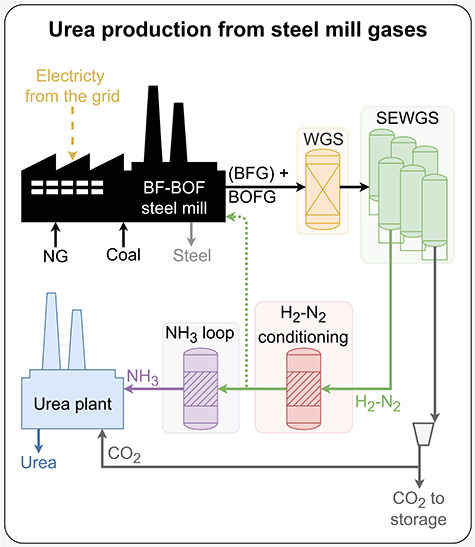

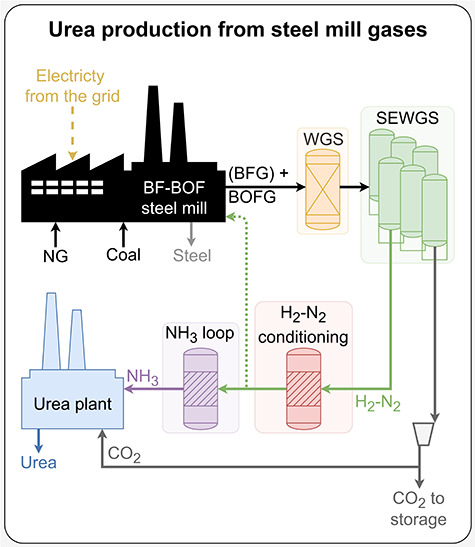

## 1 Introduction

The steel industry is a pillar of modern society and is among one of the most energy-intensive and carbon-emitting industries, accounting for approximately 8% of global energy demand and 7% of total CO_2_ emissions from the energy system.^1^ The blast furnace–basic oxygen furnace (BF–BOF) process, which uses iron ore as the main input, accounts for 90% of the primary production. Another primary production method is the direct reduced iron-electric arc furnace (DRI-EAF) route, which differs from the blast furnace–basic oxygen furnace route by using high-quality DR-grade pellets instead of raw iron ore. The DRI-EAF route uses hydrogen and carbon monoxide as reducing agents, predominantly generated from natural gas.^1^ In addition, scrap-based production is carried out in electric furnaces, which are significantly less energy-intensive. Alternative ironmaking processes, such as smelting reduction, have seen limited adoption due to technological and economic constraints. Despite advancements in energy efficiency, the production of each tonne of steel still results in an average of 1.4–1.9 tonnes of CO_2_ emissions.^1,2^ Carbon capture and storage (CCS) technologies are considered pivotal for reducing the CO_2_ emissions of the iron and steel sector, and their integration in the steelmaking industry has been investigated in the last years. The carbon capture technologies considered in peer-reviewed articles can be categorized into four main groups: (i) postcombustion (chemical absorption, adsorption, membranes), (ii) looping processes (calcium looping, chemical looping, other looping processes), (iii) oxygen blast furnaces and top-gas recycling, and (iv) precombustion (chemical absorption, adsorption, membranes, sorption enhanced water gas shift).^3^ Alongside the CO_2_ capture and storage, CCUS is gaining attention giving the possibility of decarbonizing the steelmaking sector while synthesizing an additional product. Table 1 gives an overview of recent CCU­(S) applications in the steel industry.^4^ The FReSMe project (From Residual Steel Gases to Methanol), started in 2016, demonstrated the feasibility of producing methanol from carbon dioxide, recovered via sorption-enhanced water gas shift (SEWGS) from industrial blast furnace gas (BFG), and hydrogen, which was generated both from BFG via SEWGS and by electrolysis.^5^ SEWGS is a technology that converts syngas into streams of H_2_ and CO_2_, making it particularly suitable for precombustion CO_2_ capture.^6,7^ SEWGS operates as a type of pressure swing adsorption (PSA) process, where the produced CO_2_ is in situ adsorbed on solid materials (typically potassium-promoted hydrotalcites) at temperatures between 350 and 550 °C while the WGS reaction takes place. With respect to the conventional way of producing H_2_ via steam methane reforming, which involves the use of high- and low-temperature WGS reactors and a PSA unit for the purification step, the SEWGS technology offers the possibility to combine the low-temperature WGS reactor and the PSA unit, reducing the number of process steps and required equipment.^15^ Gentile et al. performed a techno-economic assessment of the FreSMe concept, highlighting the advantages brought by industrial symbiosis and analyzing different sizes of the methanol production section.^16^ Iron and steel companies have recently started the deployment of CCUS strategies. In September 2018, ThyssenKrupp produced methanol from BFG in its steel mill in Duisburg, Germany.^4,8^ Beijing Shougang Langze New Energy Technology Co., Ltd., Baosteel, and Arcelor Mittal employed the LanzaTech biotechnology for ethanol production starting from steel gases.^4^ Baosteel’s 300 tons per year ethanol pilot plant became fully operational in March 2012 ^9^, while Arcelor Mittal demonstrated ethanol production in Steelanol and Carbon2Value projects. ArcelorMittal took part in the Steel2Chemicals project with the aim to synthetize naphtha using residual gases from steel production as feedstock.^13^ Baosteel also investigated the possibility of using microalgae for carbon sequestration and concurrent wastewater purification.^4^ Gas streams generated in a BF–BOF steel mill which contains not only CO, CO_2_ but also N_2_ can be used for microalgae growth, consequently avoiding the emissions in the atmosphere of CO_2_. In addition, CO_2_ can potentially be used in the steelmaking process to replace gases such as N_2_ and Ar. Captured CO_2_ could be used for top or bottom blowing of the converter, for the mixing of molten steel, as well as a reactant or protecting gas.^4,14^ Top-blowing CO_2_ in the converter was successfully applied by Shougang Group.^14^ In Table 1, these multiple uses of CO_2_ are labeled as “CO_2_ utilization in the steelmaking process”.

**Table 1:** CCU­(S) Applications in the Steel Industry

Application	Project	Steel company	Year
Methanol production^5^	FReSMe	Arcelor Mittal	2016
Methanol production^4,8^	Carbon2Chem	ThyssenKrupp	2018
Ethanol production^4,9^		Baosteel	2012
Ethanol production^4,10^		Beijing Shougang Langze New Energy Technology Co., Ltd.	2015
Ethanol production^4,11^	Carbon2Value	Arcelor Mittal	2019
Ethanol production^4,12^	Steelanol	Arcelor Mittal	2022
Naphtha production^13^	Steel2Chemicals	Arcelor Mittal	2018
Microalgae carbon sequestration^4^		Baosteel	2014
CO_2_ utilization in steelmaking process^4,14^		Shougang Group	
CO_2_ utilization in steelmaking process^4^		Ansteel	
CO_2_ utilization in steelmaking process^4^		Baosteel	

In addition to the steel sector, the fertilizer sector contributes significantly to global greenhouse gas (GHG) emissions. In 2018, the synthetic nitrogen fertilizer supply chain accounted for 2.1% of global GHG emissions.^17^ This figure includes emissions from production and transportation (0.9% of global anthropogenic GHG emissions) and use-related emissions (1.2%).^17^ The global ammonia and urea sectors are critical to modern agriculture and various industrial applications, playing pivotal roles in meeting global food demands and supporting industrial processes. The production processes for ammonia and urea, however, are energy-intensive and have substantial environmental impacts. The Haber–Bosch process alone is responsible for approximately 1–2% of global energy consumption and for a significant share of greenhouse gas emissions, primarily CO_2_.^18^ This has prompted the industry to explore more sustainable production methods, including advancements in carbon capture and utilization (CCU) technologies. Efforts to improve the sustainability of the ammonia and urea sectors include the development of green ammonia produced using renewable energy sources such as wind or solar power to generate the hydrogen needed for the Haber–Bosch process. This approach not only reduces reliance on fossil fuels but also significantly lowers CO_2_ emissions.^19^ On the other hand, industrial symbiosis can offer similar opportunities. Industrial symbiosis is a concept derived from biology, where different species collaborate in symbiosis for mutual benefit. Similarly, in industrial settings, synergy between different industries can lead to significant advantages, including reduced emissions, minimized waste, and enhanced energy efficiency through the exchange of resources such as water, energy, materials, and expertise.^20^ Recognized as a solution for reducing greenhouse gas emissions, improving circularity, and enhancing environmental sustainability, industrial symbiosis is a key element in the EU’s sustainable industry policy and in the Green Deal.^21^ Its popularity has surged in recent years, as evidenced by the increasing number of publications since 2007 ^20^. A systematic review by Neves et al.^20^ shows that the majority of research in this field originates mainly from China and Europe, followed by the U.S. Industrial symbiosis cases especially involve chemical, cement, iron and steel, pulp and paper industries, power plants, and refineries, being highly energy- and CO_2_-intensive sectors.^20^

This study performs a techno-economic assessment of the industrial symbiosis between the steel and fertilizer sectors through the INITIATE process. Processed steel gases, such as blast furnace gas (BFG) and basic oxygen furnace gas (BOFG), are fed to the SEWGS columns where CO_2_ is adsorbed and an H_2_–N_2_ stream is produced. This stream is then used to produce ammonia, which is subsequently converted into urea. The CO_2_ captured by SEWGS is partly used for urea synthesis. This study was carried out within the context of the INITIATE project, which aims to demonstrate the INITIATE process under real industrial conditions by employing a multicolumn SEWGS system at the Swerim AB site in Luleå, Sweden. The SEWGS technology will be demonstrated at TRL 7, building upon the experience gained in the previous STEPWISE^22,23^ and FReSMe^5,16^ projects. The key performance indicators of steel and urea symbiotic production were calculated and compared against the base and reference cases, where these goods are produced separately.

The paper is organized as follows: Section 2 introduces the methodology adopted for the techno-economic assessment of the INITIATE process; Section 3 describes the analyzed plants; Section 4 gives details about the modeling of the plants, as well as the assumption for the economic analysis and the definition of KPIs; Section 5 shows the results; and Section 4 presents the conclusions. Additional details about the methodology and the results are given in the Supporting Information.

## 2 Overall Approach

The methodology adopted for the techno-economic assessment of the INITIATE plants is outlined as follows.

1. Identification of base BF–BOF, ammonia, and urea plants in terms of size and technology. The base plants represent state-of-the-art conventional configurations. Subsequently, reference plants were established by integrating benchmark carbon capture technologies into the base layouts (Figure 1). Amine-based chemical absorption technologies were selected due to their commercial availability. Monoethanolamine (MEA), which shows a higher reactivity with CO_2_ than methyldiethanolamine (MDEA), is typically used for postcombustion CO_2_ capture, where the CO_2_ concentration is relatively low. In contrast, for precombustion processes, where the CO_2_ concentration in the gas stream is higher, MDEA is preferred because it requires less energy for regeneration.^24^ Therefore, MEA postcombustion CO_2_ capture technology was integrated in reference ammonia plants to decarbonize the flue gas of the fired tubular reformer, while the MDEA precombustion CO_2_ capture system was adopted in the reference steel plant.
2. Implementation of the industrial symbiosis concept within the INITIATE plants, maintaining the same plant sizes as the base and reference cases (Figure 1). The small-scale INITIATE plant produces 224 t_urea_/day, treating only BOFG in the SEWGS columns. For the large-scale plant, urea production is set at 1500 t_urea_/day, with both BFG and BOFG streams fully utilized by the SEWGS technology.
3. Performance simulation of SEWGS and evaluation of key performance indicators, focusing on purge and rinse steam consumption. The detailed model determines the composition and mass flow rates of product streams (CO_2_-rich and H_2_–N_2_ mixtures), as well as the sizing of the SEWGS columns, the number of columns per train, and the number of trains.
4. Comprehensive simulation of the INITIATE plants using Aspen Plus, incorporating all necessary equipment for ammonia synthesis (WGS reactors, SEWGS units, methanator, compressors, ammonia reactor, etc.). This step also includes the design of the heat exchanger network, which optimizes the recovery of waste heat to generate steam required by both the WGS reactors and the carbon capture process. Aspen Plus outputs provide data on electricity consumption, supplementary fuel requirements when waste heat is insufficient, and equipment sizing.
5. Economic evaluation of the base, reference, and INITIATE plants to complete the techno-economic analysis, calculating relevant key performance indicators for comparative assessment.

**Figure 1:**
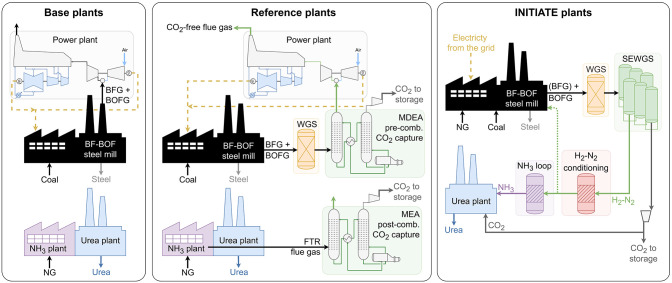
Simplified layout of the base, reference, and INITIATE plants.

## 3 Case Study

Table 2 gives an overview of the analyzed plants which are explained more in detail in the following sections. As aforementioned, this study considers two sizes of fertilizer plants: (i) the “small-scale” ammonia plant producing 128 t_NH_3__/day (43.8 kt_NH_3__/y) and suitable for the coupling with a 224 t_urea_/day (76.6 kt_urea_/y) urea plant and (ii) a “large-scale” ammonia plant plant (849 t_NH_3__/day equivalent to 290.2 kt_NH_3__/y) for the production of 1500 t_urea_/day (513.6 kt_urea_/y). On the other hand, the size of the steel plant (3.16 Mt_HRC_/y) is kept constant among all of the cases. In Europe, the size of BF–BOF steel mills varies between 1.2 and 11.5 Mt_steel_/y depending on the number of blast furnaces, with 24 sites active in 2019.^25^ Of these, 19 sites have capacities between 1.2 and 6 Mt_steel_/y.^25^ The size of the small-scale urea plant corresponds to the amount that can be produced using all of the available BOFG as feedstock. In fact, the BOFG generated in the steel plant limits ammonia production to 128 t_NH_3__/day, which translates into approximately 224 t_urea_/day. On the other hand, the size of the large-scale urea plant was set at 1500 t_urea_/day corresponding to the average plant size worldwide (in 2011, about 55% of urea plants had a capacity of 1000 t_urea_/day or less and approximately 90% had a capacity of 2000 t_urea_/day or less).^26,27^ As the total amount of NH_3_ and therefore urea that could theoretically be produced from all of the available BFG and BOFG is higher than this value, the H_2_–N_2_ excess from SEWGS not used in ammonia synthesis can instead be redirected as fuel within the steel mill.

**Table 2:** Overview of the Analyzed Plants

Plant		Product	Size	Technology	Application	CO_2_ capture
Base	Steel	HRC	9249 t_HRC_/day	BF–BOF	Steel market	None
	Ammonia	Ammonia	128 t_NH_3__/day	NG steam reforming	Coupled with urea plant	None
			849 t_NH_3__/day			None
	Urea	Liquid urea	224 t_urea_/day	Conventional	AdBlue®	None
			1500 t_urea_/day	CO_2_ stripping	Liquid fertilizer	None
Reference	Steel	HRC	9249 t_HRC_/day	BF–BOF	Steel market	MDEA precomb.
	Ammonia	Ammonia	128 t_NH_3__/day	NG steam reforming	Coupled with urea plant	MEA postcomb.
			849 t_NH_3__/day			
	Urea	Liquid urea	224 t_urea_/day	Conventional	AdBlue®	None
			1500 t_urea_/day	CO_2_ stripping	Liquid fertilizer	None
INITIATE	Steel + urea	HRC	9249 t_HRC_/day	BF–BOF	Steel market	SEWGS
		Liquid urea	224 t_urea_/day	Conventional	AdBlue®	
		HRC	9249 t_HRC_/day	BF–BOF	Steel market	
		Liquid urea	1500 t_urea_/day	CO_2_ stripping	Liquid fertilizer	

### 3.1 Base and Reference BF–BOF Plant

The blast furnace–basic oxygen furnace (BF–BOF) steel plant used as base case in this study was modeled using as reference the plant described in the IEAGHG 2013 technical report on steel plants,^28^ introducing some modifications such as the plant size and slight variations in the composition of BFG, whose composition is provided in Table 3. All the sections of the steel mill have been modeled defining the gas distribution within the steel plant and the composition of the flue gases, which is fundamental for the integration of carbon capture technologies and to carry out the techno-economic analysis.

**Table 3:** BFG, BOFG, and COG Composition

	Composition [%mol]						
Gas stream	CH_4_	CO	CO_2_	H_2_	H_2_O	O_2_	N_2_
BFG	-	21.79	20.54	2.30	4.00	-	51.36
BOFG	-	56.92	14.44	2.64	12.16	-	13.84
COG	23.24	3.87	0.97	60.05	3.15	0.19	5.82

The reference steel mill adopted in this study integrates a MDEA precombustion carbon capture section with the aim of decarbonizing the BFG + BOFG mixture, which is used in the base steel plant as fuel in the power plant. In general, in a steelmaking facility, there are numerous emission points, but the flue gas from the power plant represents roughly 50% of the CO_2_ emissions of a steel mill.^29,30^ The same reference steel mill and methodology used by Zecca et al.^31^ are considered in this study. Figures S3 and S4 in the Supporting Information give an overview of the gas distribution within the base and reference steel plants.

### 3.2 Base and Reference Ammonia Plants

Approximately 80% of globally produced ammonia is used in the fertilizer industry, with the remainder serving various industrial sectors.^32^ Ammonia is synthesized from nitrogen and hydrogen. Nitrogen is obtained from air, while hydrogen is primarily derived from fossil fuels as methane steam reforming technology accounts for 77% of global ammonia production.^32^ In steam reforming-based ammonia synthesis, the process comprises seven main steps: (i) natural gas desulfurization, (ii) catalytic steam reforming to produce hydrogen and introduce nitrogen, (iii) water gas shift reaction to convert CO into CO_2_ and generate additional hydrogen, (iv) carbon dioxide removal, (v) methanation to remove residual traces of CO and CO_2_, (vi) deep water removal, and (vii) ammonia synthesis to produce anhydrous ammonia. While all plants follow this general process, parameters such as operating pressures, temperatures, and feedstock quantities are site specific. Operating pressure of ammonia synthesis varies between 100 bar and 300 bar.^32–34^ Ammonia synthesis occurs in an iron catalyst-based converter. The NH_3_ reactor outlet gas is cooled in ammonia chillers to condense ammonia. Thus, ammonia is separated from the unreacted gas, which is recycled back to the ammonia reactor. Liquid ammonia can be stored at −33 °C and 1 atm or sent as warm product to a urea plant (10–20 °C, 10–20 bar).^32,33^ Heat generated in various plant sections, such as the reformer, shift converter, and ammonia synthesis converter, is typically utilized to generate high-pressure steam, which is expanded in steam turbines for driving the synthesis gas compressor. At the medium-pressure level, steam is extracted and used as process steam in the reforming reaction or for driving other equipment. Modern ammonia plants often export surplus steam to other consumers.^32^ The reference ammonia plants are the same size as the corresponding base ammonia plants. In these plants, flue gas from the fired tubular reformer undergoes treatment in a postcombustion carbon capture section where CO_2_ is removed using monoethanolamine (MEA) and subsequently sent to storage (Figure 1). Similarly, the CO_2_ stream from the CO_2_ removal section of the ammonia plant is also directed to storage in the case of not being used for urea production. A detailed description of the postcombustion carbon capture section used in this study is discussed by Zecca et al.^35^

### 3.3 Base Urea Plants

Urea is synthesized from ammonia and carbon dioxide, both produced in the ammonia plant. Ammonia and carbon dioxide react at high pressure forming ammonium carbamate (NH_2_COONH_4_), which then dehydrates forming urea (NH_2_CONH_2_) and water.^36^ The two reactions occur in the same reactor: the first reaction, which is fast and exothermic, goes to completion rapidly, while the second reaction, which is slower and endothermic, does not reach completion.^36^ Various processes have been developed: (i) conventional process without stripping; (ii) CO_2_ stripping process, e.g., by Stamicarbon or Toyo’s ACES process; (iii) NH_3_ stripping process, e.g., by Snamprogetti; and (iv) Isobaric Double Recycling process (IDR), applying stripping with NH_3_ and CO_2_, by Montedison.^32^ As base cases, a conventional process without stripping has been selected for the small-scale urea plant, while a CO_2_ stripping process has been chosen for the large-scale urea plant. For both plants, the final product is in liquid form, so the prilling or granulation section is not considered in this study. The steam input for the small-scale plant, based on a conventional total recycling process, is assumed to be double the amount needed for the large-scale plant. In fact, steam consumption typically ranges between 1.6 and 1.8 t_steam_/t_urea_ for the conventional total recycling process, while it reduces to 0.77–0.9 t_steam_/t_urea_ for CO_2_ stripping processes.^32^

### 3.4 INITIATE Plants

In the INITIATE process, steel gases are used as feedstock for ammonia production. First, BOFG (small-scale plant) or BFG + BOFG (large-scale plant) streams are compressed to the operating pressure of the water gas shift reactors where CO is converted to CO_2_ by adding steam and thus producing H_2_. Two WGS reactors are utilized, with only 50% of the total gas mixture fed into the first reactor.^37^ A split configuration, as proposed by Carbo et al.,^37^ was adopted to minimize steam consumption while reducing the CO content to below 5%mol in the gas stream sent to SEWGS. Consequently, the resulting H_2_O/CO ratio at the inlet of the first WGS reactor is 3, whereas at the inlet of the second reactor, it is equal to 2.^36^ The overall H_2_O/CO ratio is 1.56. The WGS reaction also takes place in the SEWGS columns, together with CO_2_ adsorption. Two streams are then available from SEWGS, one CO_2_-rich, which after being dehydrated can be used for urea production, geologically storing CO_2_ in excess, and a H_2_–N_2_ mixture, which is sent to the ammonia synthesis section. The nitrogen required for ammonia synthesis is not externally supplied but is inherently present in BOFG and BFG (Table 3), thereby eliminating the need for an additional nitrogen source. A methanator is also included in the process to convert CO and CO_2_, which are poisonous for the catalyst of the ammonia reactor, to CH_4_.

#### 3.4.1 Small-Scale INITIATE Plant

In the small-scale INITIATE plant configuration (Figure 2), all basic oxygen furnace gas available in the steel plant is processed in the WGS and SEWGS section. The production of urea is limited to the same amount as in the small-scale base and reference cases. Consequently, BOFG is no longer available for power production. Additionally, the compression of BOFG to the SEWGS operating pressure, along with the electrical consumption of all necessary equipment for ammonia and urea synthesis, increases the overall electricity demand. To meet this demand, natural gas is imported and used along with blast furnace gas in the steel plant’s power plant. Conversely, all other operations within the steel plant remain unaffected since the BOFG in the base steel plant is exclusively employed as a fuel for power generation. The steam required for the water–gas shift reactors, SEWGS, and the urea plant is generated by exploiting the heat available within the plant and using the purge gas from the ammonia loop as fuel. Main specifications of the streams shown in Figure 2 are given in Table S5 and in the Supporting Information.

**Figure 2:**
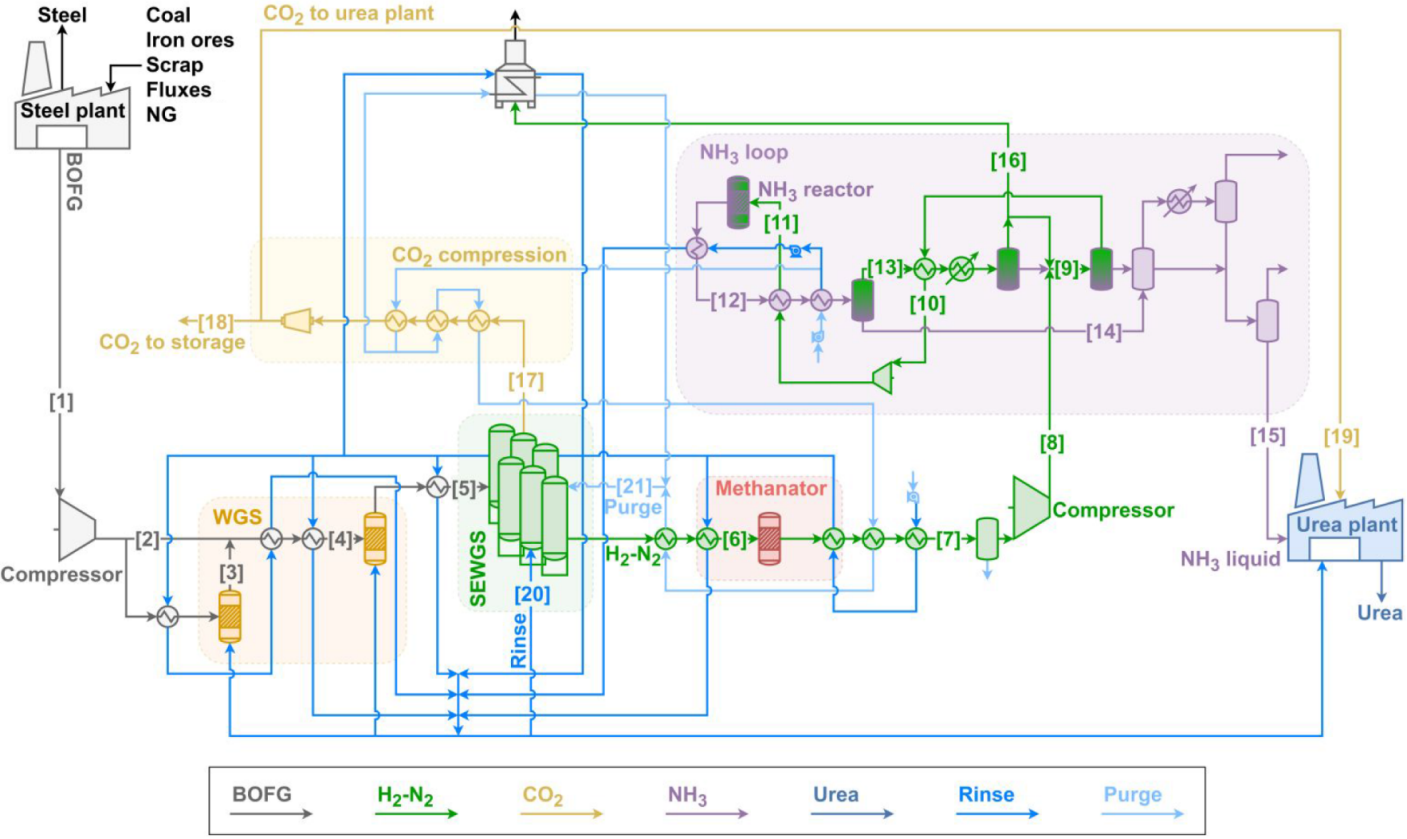
Small-scale INITIATE plant layout.

#### 3.4.2 Large-Scale INITIATE Plant

In the large-scale INITIATE plant configuration (Figure 3), the whole blast furnace gas and basic oxygen furnace gas are sent to the WGS + SEWGS section for decarbonization. Consequently, BFG and BOFG normally used for electricity production or to meet the heat requirements of the steel plant must be replaced. The electricity necessary to operate the steel plant is imported from the grid, and therefore, the power plant is absent. Additionally, part of the BFG normally used as fuel in the coke plant and in the hot-stoves is replaced by natural gas and by the H_2_–N_2_ mixture produced by the SEWGS.

**Figure 3:**
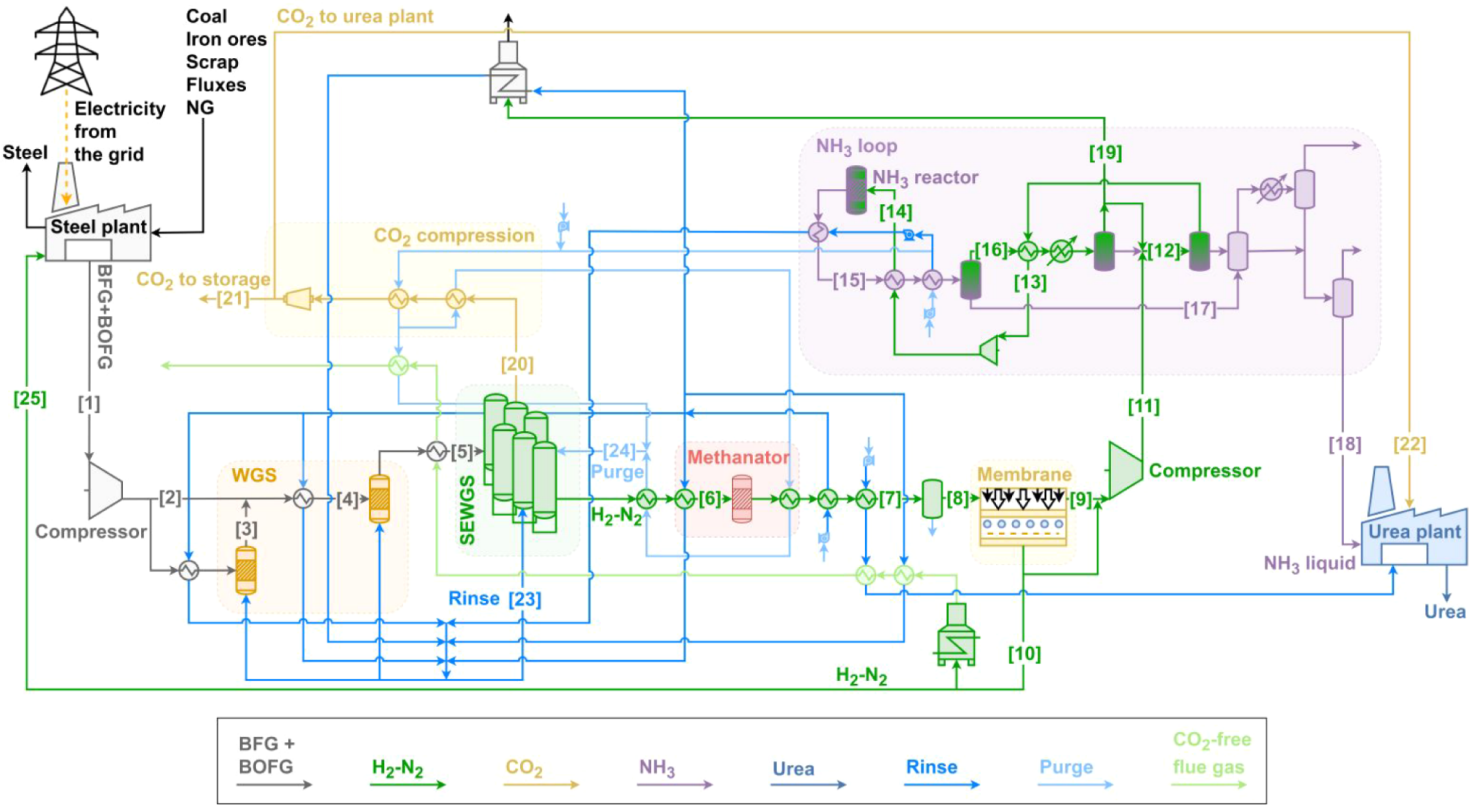
Large-scale INITIATE plant layout.

In the large-scale INITIATE plant, a membrane is integrated in the process to maintain, in the gas stream fed to the ammonia synthesis loop, the appropriate hydrogen-to-nitrogen ratio for the ammonia production process. While the small-scale plant does not require this equipment, since the ratio of CO (converted into H_2_ in the WGS stages) to N_2_ in BOFG is close to stoichiometric conditions of NH_3_ synthesis, the large-scale plant’s BFG + BOFG mixture has a lower CO to N_2_ ratio, necessitating the removal of excess nitrogen. The mass flow rate of the H_2_–N_2_ mixture sent to the ammonia loop is set to produce 849 tonnes of NH_3_ per day, suitable for a production of 1500 tonnes of urea per day. Part of the H_2_–N_2_ mixture that is not used for ammonia production is burnt to produce medium pressure (MP) steam, while the remainder is used to meet some of the heat demands of the steel plant. Additionally, the necessary steam for the water–gas shift reactors, SEWGS, and urea plant is produced by utilizing the available heat within the plant and using the purge gas from the ammonia loop as fuel. For details regarding the stream shown in Figure 3, refer to Table S6 in the Supporting Information.

## 4 Method

This section details the modeling of the plants discussed above, which were simulated in Aspen Plus V14. The property methods and the components used in Aspen Plus for modeling are summarized in Table S7 in the Supporting Information.

### 4.1 Ammonia Plant Modeling

Simulation of the ammonia plants was conducted in Aspen Plus V14 using the RKS-BM method, except for the CO_2_ capture section, where the ELECNRTL method was applied. The ammonia reactor is modeled using 4 RPlug reactors, with a Fortran subroutine linked to the Aspen model to describe the kinetics of ammonia synthesis reaction. The methodology and the kinetic model presented by Nielsen were used (eqs 1–4):^38^ eq 1 is used to calculate the reaction rate of the ammonia synthesis reaction, where *k*_eq_ (eq 2) and *K*_a_ (eq 3) are the equilibrium and adsorption constants, while α is a parameter used to correlate kinetic data.

1 $$r_{NH_{3}}\left[\frac{{\mathrm{kmol}}_{NH_{3}}}{m^{3}h}\right]=3.945\times 10^{10}e^{\left(-5622/T\right)}\frac{k_{eq}^{2}a_{N_{2}}-\frac{a_{NH_{3}}^{2}}{a_{H_{2}}^{2}}}{{\left(1+K_{a}\frac{a_{NH_{3}}}{a_{H_{2}}^{1.523}}\right)}^{2\alpha }}$$

2 $$\log_{10}k_{eq}=-2.691122\times \log_{10}T-5.519265\times 10^{-5}T+1.848863\times 10^{-7}T^{2}+2001.6/T+2.6899$$

3 $$K_{a}=2.94\times 10^{-4}e^{\left(12104/T\right)}$$

4 α=0.654

where *R* = 0.082057 L·atm·K^–1^·mol^–1^ and *T* and *P* are expressed in [K] and in [atm], respectively. The activities of the species (*a*_i_) used in eq 1 are computed with eq 5 using the values given in Table S11 in the Supporting Information.^38^

5 $$a_{i}=x_{i}P\exp\left[\frac{P}{RT}\left(B_{i}-\frac{A_{i}}{RT}-\frac{C_{i}}{T^{3}}+{\left(A_{i}^{0.5}-\sum x_{i}A_{i}^{0.5}\right)}^{2}\right)\right]$$

Additional assumptions adopted for the simulations are listed in Table S7 in the Supporting Information. Efficiency of equipment in small-scale plants is assumed to be lower compared to that in large-scale plants; for instance, process air compressors, syngas compressors, NH_3_ synthesis compressors, and CO_2_ compressors in the cleanup section have a polytropic efficiency of 0.75 for small-scale plants and 0.85 for large-scale plants. Pump hydraulic efficiency is assumed equal to 0.75 for both scales, while mechanical efficiency for all equipment is fixed at 0.95. In small-scale ammonia plants, all equipment is electrically driven, requiring electricity imported from the grid. In contrast, large-scale ammonia plants use steam turbines to drive major equipment like compressors, utilizing steam generated onsite. Consequently, steam export is higher for small-scale plants than for large-scale ones. However, when an ammonia plant is integrated with a urea plant, overall steam import is either zero or nearly zero.

### 4.2 Urea Plant Modeling

The urea plants are simulated in Aspen Plus V14 using the SR-POLAR method. The electric input for urea plants is equal to 20 kWh/t_urea_, while the steam import for the large-scale plant corresponds to 2.20 GJ/t_urea_.^39^ The urea reactor is modeled using an RPlug reactor, with a Fortran subroutine linked to the Aspen model to describe the kinetics of the reactions occurring in it. As mentioned above, the reaction of carbamate formation is exothermic and fast, reaching equilibrium, while its dehydration to urea is endothermic and does not reach equilibrium in the reactor due to its slow rate. The reaction rates of these two reactions were formulated through eqs 6 and 8.

6 $$r_{CARB}\left[\frac{kmol_{CARB}}{m^{3}s}\right]=k_{\mathrm{CARB}}\left\{x_{NH_{3}}^{2}x_{CO_{2}}-\frac{x_{\mathrm{CARB}}}{K_{\mathrm{CARB}}}\right\}$$

7 $$K_{\mathrm{CARB}}=\exp\left\{\frac{-\left(G_{\mathrm{CARB}}^{0}-2G_{NH_{3}}^{0}-G_{CO_{2}}^{0}\right)}{\mathrm{RT}}\right\}{\left(\frac{P}{P^{0}}\right)}^{2}\left(\frac{\Phi_{NH_{3}}^{2}\Phi_{CO_{2}}}{\Phi_{\mathrm{CARB}}}\right)$$

8 $$r_{\mathrm{UREA}}\left[\frac{{\mathrm{kmol}}_{\mathrm{UREA}}}{m^{3}s}\right]=k_{\mathrm{UREA}}\left\{x_{\mathrm{CARB}}-\frac{x_{\mathrm{UREA}}x_{H_{2}O}}{K_{\mathrm{UREA}}}\right\}$$

9 $$K_{\mathrm{UREA}}=exp\left\{\frac{-\left(G_{\mathrm{UREA}}^{0}+G_{H_{2}O}^{0}-G_{\mathrm{CARB}}^{0}\right)}{RT}\right\}{\left(\frac{P}{P^{0}}\right)}^{2}\left(\frac{\Phi_{\mathrm{CARB}}}{\Phi_{\mathrm{UREA}}\Phi_{H_{2}O}}\right)$$

where *K*_CARB_ and *K*_UREA_ are the equilibrium constants, *T* is expressed in [K], *P* is expressed in [atm], *x*_i_ is the molar fraction of component i, *P* ^0^ is the reference pressure (1 atm), $G_{i}^{0}$ is the ideal-gas Gibbs free energy of component i at *T*, *P* ^0^, and Φ is the fugacity coefficient of component i, *R* = 0.082057 L·atm·K^–1^·mol^–1^. The rate constant *k*_CARB_ is set to a large value in order to simulate the equilibrium reached by carbamate formation reaction, while *k*_UREA_ is computed through eq 10, where *V* ^L^ is the molar volume of the liquid.

10 $$k_{\mathrm{UREA}}=15\times 10^{8}e^{\left({-10}^{8}/\left(\mathrm{RT}\right)\right)}/V^{L}$$

Similarly to ammonia plants, the polytropic efficiency of compressors was set to 0.75 for the small-scale urea plant and 0.85 for the large-scale urea plant. The hydraulic efficiency of the pumps was considered to be 0.75 for both scales, while the mechanical efficiency for all equipment was fixed at 0.95.

### 4.3 INITIATE Plants

#### 4.3.1 SEWGS Modeling

The sorption enhanced water gas shift combines the WGS reaction with in situ adsorption of CO_2_ on potassium-promoted hydrotalcite (K-HTC) that also serves as catalyst for the WGS reaction.^40–43^ During the regeneration phase, K-HTC releases relatively pure carbon dioxide. Because of the periodic loading and regeneration of the sorbent, a state-of-the-art SEWGS system includes multiple columns operating in pressure cycles similar to those in pressure swing adsorption permitting the continuous production of CO_2_-rich and H_2_-rich streams. SEWGS is usually operated at 400 °C and 10–40 bar.^15^ Following the adsorption step, a CO_2_ or steam rinse is performed to enhance the purity of the CO_2_-rich product. This step prevents efficiency loss and contamination of the CO_2_-rich product by hydrogen or other species present in the feeding gas. Typically, 1 to 3 pressure equalization steps are performed after the rinse. During these equalizations, a high-pressure column that needs to be depressurized is connected to a low-pressure column needing pressurization. The rinse gas in the high-pressure column expands, pushing the remaining syngas into the lower-pressure column. In the final depressurization or blowdown step, the column pressure is released, allowing for the regeneration of the loaded particles. This step is followed by a steam purge to maximize carbon dioxide recovery and improve sorbent regeneration. Overall, the SEWGS process efficiently converts syngas into separate streams of hydrogen at feed pressure and carbon dioxide at regeneration pressure, both at the operating temperature of the system. Consequently, the SEWGS process is ideal for precombustion CO_2_ capture, aiding in the reduction of greenhouse gas emissions. The SEWGS operation was determined using a proprietary cycle model developed in Matlab by TNO.^7,23,44^ This model simulates a specific cycle design, accounting for relevant kinetics and adsorption equilibria. It incorporates a kinetic model describing the sorption behavior of CO_2_ and H_2_O, as well as their interactions on potassium-promoted hydrotalcite-based sorbents.^45^ The sorption kinetics and capacities of CO_2_ and H_2_O were investigated through breakthrough experiments, which were carried out by using sequences of adsorption and desorption steps with various gas mixtures containing CO_2_ and H_2_O.^46^ The simulation results provide a full characterization of the cycle, including steam consumption rates, the number and sizes of columns, and the compositions of the CO_2_ and H_2_-rich product streams. A cyclic steady-state is assumed to be achieved when the mass and energy balances converge with an absolute tolerance below 10^–5^. Design criteria for the SEWGS cycle typically focus on the carbon capture ratio (eq 11), indicating the amount of captured carbon dioxide relative to the CO and CO_2_ fed, and CO_2_ purity (eq 12, which is the ratio between the mole flow rate of CO_2_ present in the CO_2_-rich stream and the total flow rate of the CO_2_-rich stream calculated on dry basis), crucial for process efficiency as any inclusion of H_2_ and CO in the CO_2_ product diminishes performance. Three key variables are evaluated: productivity (CO_2_ produced per unit time per unit of sorbent, eq 13), rinse steam consumption (steam used in rinse relative to CO and CO_2_ fed), and purge steam consumption (steam used in purge relative to CO and CO_2_ fed). SEWGS cycle design aims to minimize capital expenditure and operational energy costs by calibrating the total steam consumption and productivity, also taking into consideration the WGS reactor(s) steam demand. The SEWGS, indeed, completes the WGS reaction, allowing to limit the CO conversion in the upstream WGS stage(s) and thus reducing the steam consumption, compared to a conventional configuration, which consist of a series of WGS reactors, when the same overall CO conversion is considered.^30^ A parametric analysis was hence performed with the aim of achieving the target values for the carbon capture rate and CO_2_ purity. The results are then incorporated into Aspen Plus by using a calculator and other native equipment (Table S7 and Supporting Information for more details).

11 $$\mathrm{CCR}\left[\%\right]=\frac{{\left({ṁ}_{CO_{2}}\right)}_{CO_{2}-\mathrm{product}}}{{\left({ṁ}_{CO_{2}}+{ṁ}_{\mathrm{CO}}\right)}_{\mathrm{feed}}}\times 100$$

12 $$\mathrm{CP}\left[\%\right]=\frac{{\left({ṅ}_{CO_{2}}\right)}_{CO_{2}-\mathrm{product}}}{{ṅ}_{CO_{2}-\mathrm{product},\mathrm{dry}}}\times 100$$

13 $$\mathrm{Productivity}\left[\frac{{\mathrm{mol}}_{CO_{2}}}{kg_{\mathrm{sorbent}}s}\right]=\frac{{\left({ṅ}_{CO_{2}}\right)}_{CO_{2}-\mathrm{product}}}{m_{\mathrm{sorbent}}}$$

#### 4.3.2 N_2_–H_2_ Conditioning Unit

The H_2_–N_2_ mixture exiting the SEWGS columns is sent to a methanator reactor, with an inlet temperature of 250 °C. In the case of the large-scale INITIATE plant in which a mixture of BFG + BOFG is used as feedstock for the synthesis of ammonia, since N_2_ accounts for more than 50%mol of BFG (Table 3), the ratio between H_2_ and N_2_ in the stream exiting the methanator is significantly below the stoichiometric ratio of three required for ammonia synthesis reaction. A polymeric membrane is then incorporated into the plant layout of the large-scale INITIATE plant to achieve the desired H_2_ to N_2_ ratio (Figure 4). Membrane technology represents one of the most established and economically viable approaches for gas separation.^47^ Since 1979, it has been employed in ammonia plants for hydrogen recovery from purge gas streams.^47^ The use of membranes has expanded to a wide range of industrial applications, including carbon dioxide separation from the flue gas of coal-fired power plants,^48^ hydrogen recovery in refineries,^46^ syngas ratio adjustment,^46^ air separation, and gas dehydration.^47^ In comparison with alternative separation technologies such as pressure swing adsorption and cryogenic distillation, membrane-based systems offer several advantages, including higher energy efficiency, a superior surface area-to-volume ratio, and a reduced environmental impact.^49^ A 1D model of a membrane was thus developed to calculate the necessary membrane area to achieve the desired composition of the gas stream fed to the ammonia synthesis loop. The membrane model, combined with economic data related to the capital expenditure of the membrane, was used to determine the permeate pressure that minimized the total cost. Decreasing the permeate pressure reduces the membrane area and thus the CAPEX, but it increases the cost of the subsequent compression required for ammonia synthesis. In this process, the permeate (almost pure H_2_) is mixed with part of the retentate (which composition is around 80% N_2_ and 20% H_2_) to reach the stoichiometric ratio of three between H_2_ and N_2_ in the gas mixture sent to the ammonia synthesis loop (Figure 4). The permeability of hydrogen was computed using eq 14,^50^ commonly referred as Richardson’s equation.^51^

14 $$J_{H_{2}}\left[\frac{\mathrm{mol}}{s}\right]=P_{H_{2}}^{0}/\delta e^{\left(-\frac{E_{a}}{\mathrm{RT}}\right)}\left[P_{H_{2},\mathrm{retentate}}^{n}-P_{H_{2},\mathrm{permeate}}^{n}\right]$$

where $P_{H_{2}}^{0}/\delta$ [mol·m^–2^·s^–1^·Pa^–1^] is the hydrogen permeance, *E*_a_ is the activation energy [J·mol^–1^], *R* is the ideal gas constant [J·mol^–1^·K^–1^], *T* is the absolute temperature of system [K], and δ is the membrane thickness [m]. Based on the findings of Lu et al.,^49^ the value of $P_{H_{2}}^{0}/\delta$ ranges between 10^–9^ and 3·10^–7^ mol·m^–2^·s^–1^·Pa^–1^. For this study, an intermediate value of 10^–8^ mol·m^–2^·s^–1^·Pa^–1^ was chosen. The activation energy *E*_a_ was set to 0 J·mol^–1^, while the exponent “*n*” is equal to 1. For each pressure level of the permeate side considered in the sensitivity analysis conducted, the membrane area to obtain a flow of 3368 kmol/h of H_2_ and 1123 kmol/h of N_2_ fed into the ammonia synthesis loop was computed. According to the model, the maximum permeate pressure to reach the target is 3.10 bar. A sensitivity analysis was performed by varying the electricity price between 50 €/MWh and 250 €/MWh and the permeate pressure between 1 and 3 bar. The power consumed by the compressor downstream of the membrane, necessary to increase the gas stream pressure from the permeate pressure to 312 bar (which is the design pressure of the gas sent to the ammonia synthesis loop), was computed using Aspen Plus V14, simulating a 4-stage intercooled compressor, with a cooling temperature of 15 °C and polytropic and mechanical efficiencies of the stages equal to 0.85 and 0.95, respectively. The syngas compressor employed in INITIATE plants is a centrifugal compressor as used in ammonia plants.^32^ The capital cost of the membrane was calculated by assuming a fixed charge factor (FCF) of 9.37% over 25 years (the assumed lifetime of the plants), a membrane lifetime of 5 years, and a cost of the membrane equal to 50 €/m^2^.^52^ The cost associated with the electricity consumed by the compressor along with the membrane CAPEX were used to compute the total annual cost associated with the membrane and to determine the most cost-effective pressure level for the permeate side. Figure 5 shows the total annual costs for different values of permeate pressure and electricity prices. The results indicate that for an electricity price of 50 €/MWh, the minimum cost occurs when the permeate pressure is 1 bar. However, as expected, when the electricity price increases, the minimum shifts toward higher values of the permeate pressure. Since in most cases, the minimum cost is reached when the permeate pressure is 2 bar, this value was selected.

**Figure 4:**
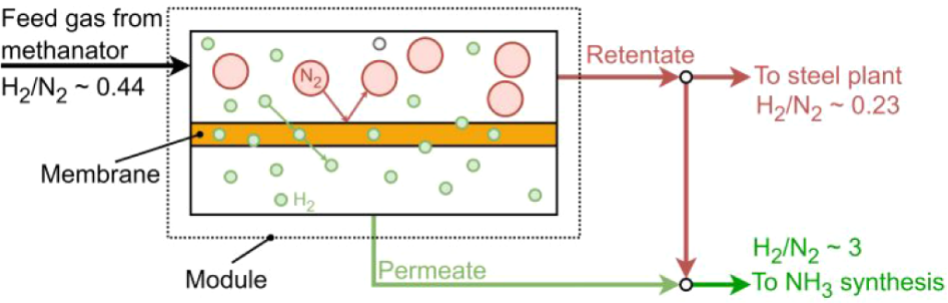
Schematic of the polymeric membrane adopted in a large-scale INITIATE plant for gas conditioning.

**Figure 5:**
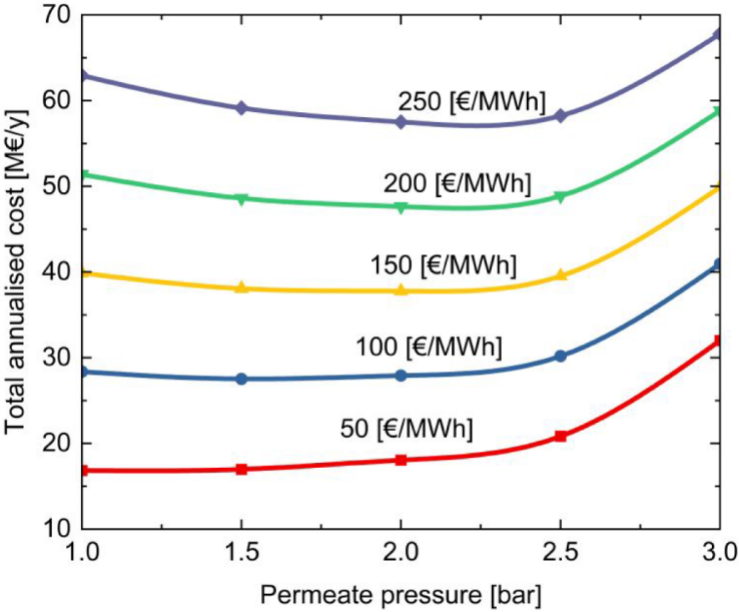
Membrane total annualized cost. Each color represents a different value of electricity price.

### 4.4 Techno-Economic Assessment Methodology

General assumptions used in the techno-economic assessment and common to all the plants analyzed are shown in Table 4. The CO_2_ emissions associated to electricity imported from the grid are set to 250 kg_CO_2__/MWh. This value is slightly higher than the average CO_2_ intensity of electricity generation in the European Union in 2023 reported at 210 kg_CO_2__/MWh.^53^ Nevertheless, a sensitivity analysis varying this parameter was performed. Water consumption in the base steel, ammonia, and urea plants was quantified using literature data,^28,33^ to which the contribution of benchmark CO_2_ capture sections (estimated from the values listed in Table 4) was added to calculate the total water consumption of the reference plants. In the case of the INITIATE plants, water consumption accounts for the requirements of the steel plant, the steam demand of the WGS reactors, and of the SEWGS unit and for cooling purposes.

**Table 4:** Assumptions Common to All Plants for the Techno-Economic Assessment

Parameter	Unit	Value
Reference CO_2_ footprint electricity from the grid	kg_CO_2__/MWh_el_	250
NG LHV	MJ/kg	46.87
Currency exchange	€/$	0.92
Reference natural gas price	€/MWh_LHV_	50
Reference electricity price	€/MWh_el_	125
Electricity selling price	€/MWh_el_	1/3·El. price
Discount rate^33,54^	%	8
Plants lifetime	years	25
Plants availability	h/y	8200
Fixed charge factor	%	9.37
Water cost	€/m^3^	1.5
CO_2_ transport and storage	€/t_CO_2__	40
CO_2_ tax	€/t_CO_2__	0
Personnel annual salary	€/y	60000
N° additional employees per CC section	-	15
Maintenance cost for CO_2_ capture section	% TPC_CC section_	2.5
Chemical and CO_2_ capture plants—Total installation cost^30,54^	% TEC	104
Chemical and CO_2_ capture plants—Indirect costs^30,54^	% TDPC	14
Chemical and CO_2_ capture plants—Contingency^30,54^	% EPC	10
Chemical and CO_2_ capture plants—Owner’s costs^30,54^	% EPC	5
Ammonia plant—Number of employees	-	33
Urea plant—Number of employees	-	33
^32^Ammonia and urea plants—Indirect costs^33^	% TDPC	20
Ammonia and urea plants—Maintenance cost^33^	% TDPC	1.5
Ammonia and urea plants—Maintenance labor^33^	% Maintenance cost	40
Ammonia and urea plants—Maintenance materials^33^	% Maintenance cost	60
Ammonia and urea plants—Administration and overheads^33^	% O&M. labor	30
Ammonia and urea plants—Insurance and local taxes^33^	% TDPC	1
Ammonia and urea plants—Contingencies and owner’s costs^33^	% EPC	35
Ammonia and urea plants—Chemicals and catalyst price	€/t_product_	2.95
INITIATE—SEWGS sorbent lifetime	years	5
INITIATE—maintenance costs	% TPC_INITIATE_·FCF	5
INITIATE—cost of membrane	€/m^2^	50
INITIATE—lifetime of membrane	years	5
Make-up water in MDEA section^28^	t_H_2_O_/t_CO_2_ stored_	3.07
Make-up water in MEA section^28^	t_H_2_O_/t_CO_2_ stored_	4
MEA price^55^	€/kg	1.01
MDEA price^55^	€/kg	2.02

The methodology used to carry out the economic assessment follows the bottom-up approach widely used in techno-economic studies.^29,30,35^ Total Annual Cost (TAC) of the analyzed plants was computed starting from the Total Equipment Cost (TEC) using the updated reference costs shown in Table 5 and the methodology described by Zecca et al.^35^ The CEPCI index of June 2024 (equal to 798.8) was used to update the cost of equipment found in the literature.

**Table 5:** Equipment Reference Cost

Equipment	Scaling parameter	*C*_0_ [M€]	*S*_0_	*f*
CO_2_ capture unit (MDEA)^56^	CO_2_ mass flow rate [t/h]	10.52	12.4	0.60
CO_2_ capture unit (MEA)^30^	CO_2_ mass flow rate [kg/s]	85.25	53.7	0.80
CO_2_ compressor and condenser^30^	Power [MW]	55.24	50.5	0.67
Furnace^29^	Heat duty [MW]	0.30	1.00	1.00
Compressor^30^	Power [MW]	10.17	15.3	0.67
Pump^57^	Volumetric flow [m^3^/h]	0.28	250	0.14
WGS^30^	H_2_ and CO flow rate [kmol/s]	3.89	1.68	0.67
Gas turbine^30^	Power [MW]	62.02	272.1	0.67
Steam turbine^30^	Power [MW]	41.43	200	0.67
Heat exchanger^58^	Heat transfer [MW]	16.25	138	0.67
Cooling tower^30^	Heat rejected [MW]	62.27	470	0.67
SEWGS single train^30^	Inlet molar flow rate [kmol/s]	11.15	1.56	0.67
Ammonia reactor^59^	Ammonia production [kg/s]	12.20	17.3	0.67

To accurately determine the total plant cost for the base steel mill, data found in the IEAGHG 2013 technical report on steel plants^28^ have been used as reference and adapted to this study, taking into account the different sizes of the plants. The cost of the power plant was estimated by using the data listed in Table 5. In the case of ammonia and urea plants, the main assumptions for conducting the economic assessment were sourced from IEAGHG 2017 technical report on ammonia plants.^33^ Total direct plant costs for small-scale ammonia and urea plants were provided by an industrial partner of the INITIATE consortium. The costs for plants producing 85.8 t_NH_3__/day and 150 t_urea_/day corresponding to 55 and 40 M€, respectively, were provided.

Being the size of the small-scale ammonia and urea plants considered in this work equal to 128.3 t_NH_3__/day and 224.2 t_urea_/day, the total direct plant cost was computed through eq 15 using a scale factor equal to 0.3 as indicated within the INITIATE consortium. Conversely, costs for large-scale ammonia and urea plants were estimated using eq 15 based on data from IEAGHG 2017 technical report on ammonia plants,^33^ adjusted for the different plant sizes considered in this study (Table 6).

15 $$C_{e}=n\times C_{0}{\left(\frac{S_{e}}{n\times S_{0}}\right)}^{f}$$

**Table 6:** Parameters Used to Estimate the Total Plant Costs of Ammonia and Urea Plants

Plant	Scaling parameter	*C*_0_ [M€]	*S*_0_	*f*	*S*	*C*_e_ [M€]
Ammonia small-scale	Size [t_NH_3__/day]	55	85.8	0.30	128	62
Urea small-scale	Size [t_urea_/day]	40	150	0.30	224	45
Ammonia large-scale	Size [t_NH_3__/day]	456	1345	0.67	849	335
Urea large-scale	Size [t_urea_/day]	331	2380	0.67	1503	244

In eq 15, *S*_e_ denotes the size of the equipment for which the cost *C*_e_ must be estimated, *S*_0_ and *C*_0_ represent the size and cost of reference equipment, *n* is the number of units, and *f* is the scaling factor. Electricity and natural gas price were set to 125 €/MWh and 50 €/MWh respectively. The prices used in the assessment were computed as the average values for the European Union between 2019 and 2022.^60^ Annual industrial electricity prices, inclusive of environmental taxes and levies for very large consumers, were considered. Similarly, annual industrial natural gas prices, including taxes for large consumers, were used. Due to the high uncertainty related to the price of these commodities, a sensitivity analysis was performed, and the results are shown in Section 5.3.1. The cost of CO_2_ transport and storage was set at 40 €/t_CO_2__ which aligns with the estimates provided by Smith et al., who suggested a range between 4 and 45 $/t_CO_2__ depending on factors such as transport distance, scale (i.e., quantity of CO_2_ transported and stored), monitoring assumptions, reservoir geology, and pipeline capital costs.^61^

### 4.5 Key Performance Indicators

The comparison across all investigated cases is conducted using economic and environmental key performance indicators, commonly referenced in literature.^29,30,35^ The environmental indexes considered include the primary energy consumption (PEC), which computes the specific energy consumed accounting for the chemical energy of the fossil fuels used in the production process as feedstock (*ṁ*_fuel_LHV_fuel_) or to generate heat (*Q̇*_req_/η_th_) and the primary energy consumption associated with the electricity generation (PEC_el_), the specific CO_2_ emissions (e_CO2_), the CO_2_ capture ratio (CCR), the specific primary energy consumption per unit of CO_2_ avoided (SPECCA) which indicates the additional amount of primary energy required to avoid the emission of 1 ton of CO_2_, and CO_2_ avoidance (CA). The subscripts “no capture” and “capture” in eqs 18, 19, and 21 refer to plants without and with CO_2_ capture system integration, respectively, while in eqs 16, 17, 20, and 21, “*x*” indicate the generic product, i.e., hot rolled coil, ammonia, or urea.

16 $$\mathrm{PEC}\left[\frac{GJ_{\mathrm{LHV}}}{t_{x}}\right]=\frac{{ṁ}_{\mathrm{fuel}}{\mathrm{LHV}}_{\mathrm{fuel}}+{\mathrm{PEC}}_{\mathrm{el}}+{\mathrm{Q̇}}_{\mathrm{req}}/\eta_{\mathrm{th}}}{{ṁ}_{x}}$$

17 $$e_{CO_{2}}\left[\frac{t_{CO_{2}}}{t_{x}}\right]=\frac{{ṁ}_{CO_{2}}}{{ṁ}_{x}}$$

18 $$\mathrm{SPECCA}\left[\frac{GJ_{\mathrm{LHV}}}{t_{CO_{2}}}\right]=\frac{{\mathrm{PEC}}_{\mathrm{capture}}-{\mathrm{PEC}}_{\mathrm{no}\mathrm{capture}}}{e_{CO_{2},\mathrm{no}\mathrm{capture}}-e_{CO_{2},\mathrm{capture}}}$$

19 $$\mathrm{CA}\left[\%\right]=\frac{e_{CO_{2},\mathrm{no}\mathrm{capture}}-e_{CO_{2},\mathrm{capture}}}{e_{CO_{2},\mathrm{no}\mathrm{capture}}}\times 100$$

In this study, the primary energy consumption (PEC) associated with electricity generation varies based on its carbon intensity using the methodology and the values indicated by Zecca et al.^31^ In the case of the base BF–BOF plant, some of the electricity is exported. In addition to the revenues for the selling of electricity, reductions in emissions and primary energy consumption are considered and calculated based on the specific scenario chosen for the carbon footprint of electricity generation. CO_2_ embedded in the urea molecule is not considered as emitted and therefore is not accounted in the computation of the KPIs (i.e., $e_{{\mathrm{CO}}_{2}}$ , CA, and CCA). The economic performance is assessed by computing the levelized cost of products, hot rolled coil (LCOHRC), ammonia (LCOA), and urea (LCOU), and the cost of CO_2_ avoidance (CCA).

20 $$\mathrm{LCOX}\left[\frac{€}{t_{x}}\right]=\frac{\mathrm{TAC}}{{ṁ}_{x}\times h_{\mathrm{eq}}}\times 10^{6}$$

21 $$\mathrm{CCA}\left[\frac{€}{t_{x}}\right]=\frac{{\mathrm{LCOX}}_{\mathrm{capture}}-{\mathrm{LCOX}}_{\mathrm{no}\mathrm{capture}}}{e_{CO_{2},\mathrm{no}\mathrm{capture}}-e_{CO_{2},\mathrm{capture}}}$$

## 5 Results

### 5.1 Base and Reference Steel Plants

The BF–BOF steel mill consumes 4845 t/day of coking coal in the coke plant and 1473 t/day of PCI coal in the blast furnace, while the plant electricity needs are entirely met by the integrated combined cycle. Any excess electricity is sold. The CO_2_ direct emissions amount to 2.068 t_CO_2__/t_HRC_. Both the equivalent emissions for exporting electricity (which are subtracted from the total) and the indirect CO_2_ emissions associated with iron ore production vary depending on the carbon footprint of the electricity scenario considered. The resulting primary energy consumption, which also accounts for the export of electricity, is 21.25 GJ/t_HRC_, primarily related to coal consumption. The CO_2_ emission distribution within the base BF–BOF steel plant is detailed in Table S1 (Supporting Information), with the power plant, hot-stoves, sinter plant, and coke plant accounting for 90% of the total emissions. The consumption of coal in the reference BF–BOF steel plant remains consistent with the base plant but with a primary energy consumption increase to 22.70 GJ/t_HRC_. Integrating a precombustion carbon capture section has the effect of reducing the efficiency of the power plant (resulting in lower internal electricity generation) and of increasing the power consumption due to the compression of the gas mixture fed to the MDEA carbon capture section, as well as the compression of the CO_2_ stream for transport and storage purposes. The electricity generation in the power plant is reduced compared to that in the base BF–BOF plant due to the absence of a low-pressure steam turbine. Instead, steam from the medium-pressure steam turbine is utilized in the water gas shift reactor and for regenerating the solvent. Additionally, some steam is generated via a natural gas-fired steam generator. The total emissions amount to 1406.5 t_CO_2__/t_HRC_, resulting in a carbon avoidance rate of 33.26% compared to the base BF–BOF steel plant. Emissions processed through the carbon capture section equal 959.4 t_CO_2__/t_HRC_, with 794.4 t_CO_2__/t_HRC_ subsequently sent for storage. The gas distribution within the reference steel plant is shown in Figure S4 in the Supporting Information. By an economic point of view, the levelized cost of hot rolled coil for the base case is 528 €/t_HRC_ which increases to 604 €/t_HRC_ for the reference steel mill. Details about the economic results are given in Table 8 and in Table S3 of the Supporting Information.

### 5.2 Ammonia and Urea Plants

The main results for the base and reference ammonia plants coupled with urea plants are summarized in Figure 6. The total primary energy consumption is 22.7 GJ/t_urea_ for the small-scale base case and 19 GJ/t_urea_ for the base, large-scale plant. This difference arises from the higher energy intensity of the small-scale ammonia plant compared to the large-scale one, as well as the higher steam consumption in the small-scale urea plant. Small-scale plants import electricity from the grid, whereas large-scale plants generate it on-site. In the case of the reference plants, additional steam is required to provide the energy demand of the postcombustion CO_2_ capture process, which is integrated to decarbonize the reformer flue gas. This steam is assumed to be produced on-site in natural gas-fired boilers; therefore, the associated CO_2_ emissions from steam generation are included in the overall assessment. Considering only the urea base plants (excluding the ammonia plants), the energy intensities are 4.5 GJ/t_urea_ for the small-scale plant and 2.2 GJ/t_urea_ for the large-scale plant, which are in accordance with published data ranging from 1.7 GJ/t_urea_ to 5.5 GJ/t_urea_.^32^ The CO_2_ footprint of the process is 0.52 t_CO_2__/t_urea_ for the small-scale plant and 0.33 t_CO_2__/t_urea_ for the large-scale plant. This difference is primarily due to indirect emissions related to electricity import in the small-scale ammonia plant. More details about emissions and primary energy consumptions can be found in the Supporting Information.

**Figure 6:**
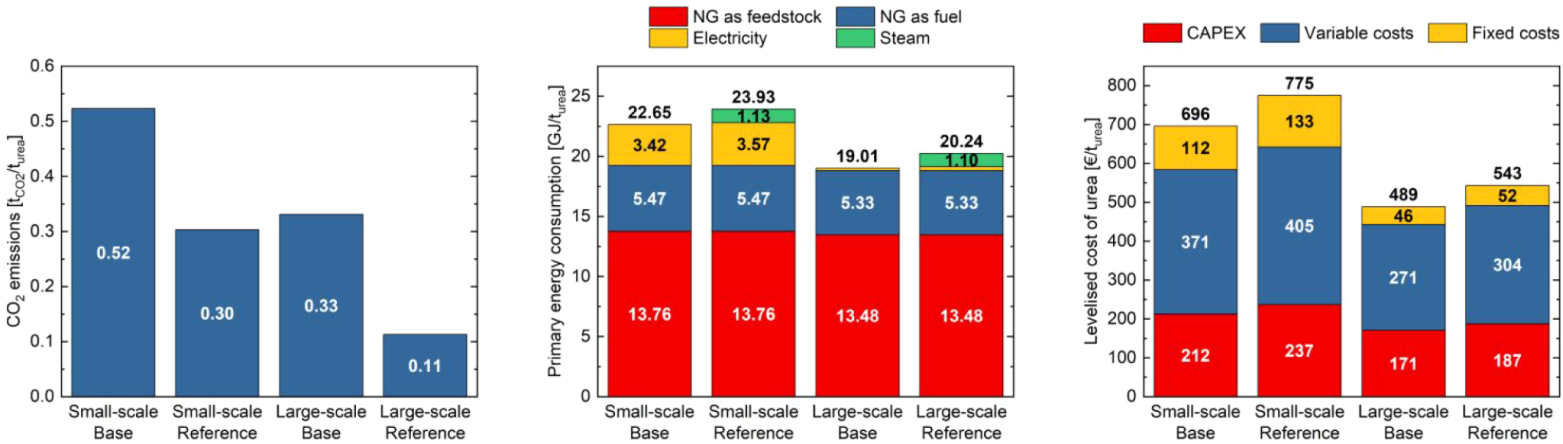
Main KPIs of the ammonia plants coupled with urea plants: carbon intensity (left), primary energy consumption (center), and levelized cost of urea (right).

In the reference plants, the primary energy consumption increases compared to the corresponding base case due to the addition of the CO_2_ carbon capture section. The SPECCA is very similar for the small- and large-scale reference plants, around 6 GJ/t_CO_2__. For the small-scale plants, the levelized cost of urea (LCOU) is 696 €/t_urea_ for the base case and 775 €/t_urea_ for the reference case. For large-scale plants, the LCOU reduces to 489 €/t_urea_ for the base case and to 543 €/t_urea_ for the reference case. The cost of CO_2_ avoided is 357 €/t_CO_2__ for the small-scale reference plant and 250 €/t_CO_2__ for the large-scale reference plant. As shown in Figure 6, CAPEX represents around 30–35% of LCOU, while variable costs contribute the most, accounting for about 55% of LCOU. Additional results can be found in Table S4 and the Supporting Information.

### 5.3 INITIATE Plants

In the small-scale INITIATE plant, some natural gas is imported and mixed with BFG to replace the BOFG normally used for power production, thereby generating all the electricity necessary to run the plant without any export (Figure S9 in the Supporting Information). In the case of the large-scale INITIATE plant, all the electricity necessary to run the plant is imported from the grid as the whole BFG + BOFG is sent to the SEWGS process. As electricity must be imported, its indirect emissions should be accounted for, and an electricity CO_2_ footprint should be assumed. Therefore, all the results will be presented under two cases: a fully renewable scenario (0 kg_CO_2__/MWh_e_) and 250 kg_CO_2__/MWh_e_. Part of the H_2_–N_2_ mixture produced by SEWGS and not used as feedstock for ammonia synthesis is combusted to generate steam, while the rest is recycled back to the steel plant to be used in the coke plant, substituting the BFG in the underfired heating, and in the hot stoves where it is mixed with NG covering most of the energy demand (Figure S10 in the Supporting Information). Details of CO_2_ emissions for the base and reference BF–BOF steel mills and the steel section of small- and large-scale INITIATE plants can be found in Table S1. Similarly, the breakdown of CO_2_ emissions for the base and reference ammonia and urea plants and the chemical section of small- and large-scale INITIATE plants are shown in Table S2. Table 7 presents the main results of the analyzed plants, regarding the import of raw materials, emissions, and key performance indicators such as SPECCA, carbon avoidance, and cost of CO_2_ avoided. In the case of base plants, indirect CO_2_ emissions and primary energy associated with electricity show negative values because the electricity exported exceeds the electricity imported. To enable a fair comparison between plants and to account for electricity export, a corresponding reduction in CO_2_ emissions and primary energy consumption associated with the production of the exported electricity is considered. Electricity export implies that the same amount of electricity does not need to be generated by alternative processes. The magnitude of the reduction in CO_2_ emissions and primary energy consumption depends on the selected energy scenario. For instance, in a renewable energy scenario, both indirect CO_2_ emissions and primary energy consumption associated with electricity export are zero. Consequently, negative values in the results should not be interpreted as CO_2_ being captured from the air and stored. The small-scale INITIATE plant achieves a carbon avoidance of 4.5%, as it processes only the BOFG from the steel plant at a cost of 24 €/t_CO_2__. For the large-scale INITIATE plant, carbon avoidance is 55.8%, with a SPECCA of 2.3 GJ/t_CO_2__ and a cost of CO_2_ avoided of 129 €/t_CO_2__. The consumption of natural gas is significantly reduced compared with the base and reference cases, while the import of electricity is increased. Consequently, indirect emissions represent a significant share of total emissions for a large-scale INITIATE plant. However, electrification serves as a primary method for reducing direct process emissions. Water consumption increases when carbon capture technologies are integrated, with INITIATE plants showing a lower raw water import compared to the reference cases. When considering a renewable energy scenario, the carbon avoidance for the large-scale INITIATE plant increases to 67.8%, whereas the carbon avoidance for all other cases is only slightly affected due to their limited import of electricity. Figure 7 illustrates the carbon avoidance and the SPECCA of both reference and INITIATE plants compared to the base plants, evaluated in relation to the carbon footprint associated with imported electricity, highlighting how different energy scenarios impact the overall environmental performance. For the reference plants, CO_2_ avoided in the small- and large-scale configurations exhibits only minor variation, as the two cases differ solely in the capacity of the ammonia and urea plants, while the steel plant size remains unchanged. Given that the CO_2_ emissions avoided in the steel plant constitute the predominant share of the total CO_2_ reductions, the overall amount of CO_2_ avoided increases only to a limited extent in the large-scale case. Although the larger ammonia plant captures a greater quantity of CO_2_ than the small-scale configuration, this additional contribution is comparatively minor relative to that of the CO_2_ captured in the reference steel plant. Consequently, the total amount of avoided CO_2_ remains almost constant across the two cases. As mentioned above, in most cases, the carbon avoidance is slightly influenced by carbon footprint of electricity, except for the large-scale INITIATE plant. SPECCA, on the other hand, reveals significant sensitivity to changes in the carbon footprint of electricity. This occurs because both the CO_2_ emissions and the total primary energy consumption of the plants depend on the electricity scenario selected. Particularly interesting is the potential for SPECCA to become negative in scenarios featuring renewable energy sources with zero carbon emissions. This outcome indicates that INITIATE plants can achieve lower primary energy consumption compared to conventional plants while reducing the CO_2_ emissions, driven by the reduced reliance on natural gas and the use of renewable electricity. Analyzing the small-scale INITIATE plant, it outperforms its corresponding reference case across various scenarios, showing a minor SPECCA. This advantage is attributed to higher imports of electricity and natural gas in the reference setup compared to INITIATE.

**Figure 7:**
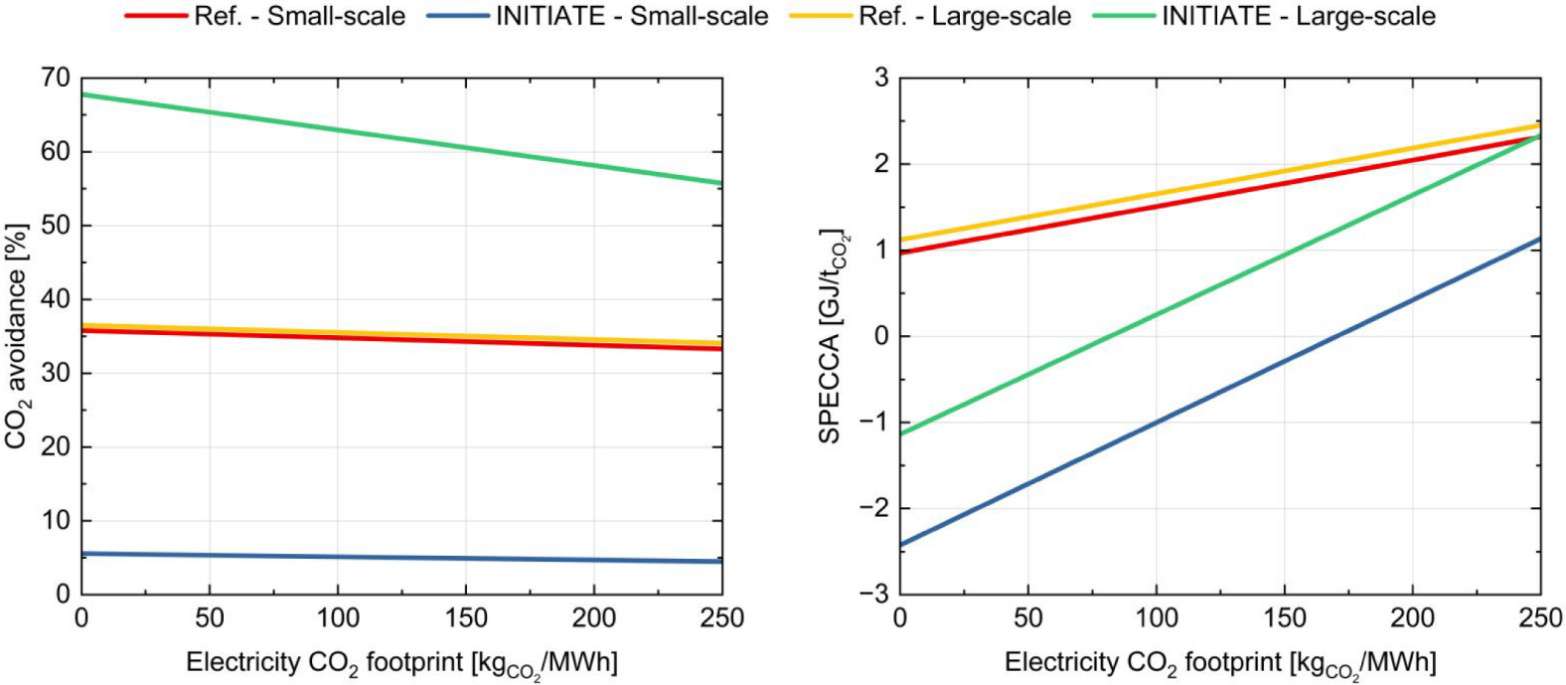
CO_2_ avoidance (left) and SPECCA (right) of reference and INITIATE plants with respect to base plants as a function of electricity carbon footprint.

**Table 7:** Main Techno-Economic Results Considering a Carbon Footprint of Imported Electricity Equal to 250 kg_CO_2__/MWh

		Small-scale			Large-scale		
Parameter	Unit	Base	Reference	INITIATE	Base	Reference	INITIATE
Coking coal import	t/day	4845.2	4845.2	4845.2	4845.2	4845.2	4845.2
PCI coal import	t/day	1472.6	1472.6	1472.6	1472.6	1472.6	1472.6
NG import	t/day	92.0	205.6	35.6	603.4	746.9	274.2
Electricity export	MW	42.87	0	0	42.87	0	0
Electricity import	MW	7.46	45.69	0	2.88	42.72	363.13
Steel production	t/day	9248.8	9248.8	9248.8	9248.8	9248.8	9248.8
Urea production	t/day	224.2	224.2	224.2	1503.3	1503.3	1503.3
Water consumption	t/day	48926.9	71744.3	49426.2	50134.3	74437.0	53001.6
CO_2_ direct emissions	t/day	19199.1	12179.9	18108.4	19607.1	12299.8	6044.2
CO_2_ indirect emissions	t/day	–212.4	274.2	0	–239.9	256.3	2178.8
CO_2_ emissions total	t/day	19608.7	13076.0	18730.4	19989.2	13178.0	8845.0
CO_2_ emissions total	Mt_CO_2__/y	6.70	4.47	6.40	6.83	4.50	3.02
CO_2_ captured^a^	t/day	-	7413.1	1108.1	1097.4	7784.4	13852.8
CO_2_ into urea product	t/day	173.8	173.8	173.8	1097.4	1097.4	1097.4
CO_2_ to storage	t/day	-	7413.1	934.3	-	7784.4	12755.4
CO_2_ capture rate^a^	%	-	85 (MDEA) 90 (MEA)	97 (SEWGS)	-	85 (MDEA) 90 (MEA)	97 (SEWGS)
PEC coking coal	MW	1756.5	1756.5	1756.5	1756.5	1756.5	1756.5
PEC PCI coal	MW	569.5	569.5	569.5	569.5	569.5	569.5
PEC NG	MW	49.9	128.6	19.3	327.3	422.2	148.8
PEC electricity	MW	–42.2	54.4	0.0	–47.6	50.9	432.3
Total PEC	MW	2333.8	2509.0	2345.3	2605.7	2799.1	2907.1
Carbon avoidance	%	-	33.32	4.48	-	34.07	55.75
SPECCA	GJ/t_CO_2__	-	2.32	1.14	-	2.45	2.34
Cost of CO_2_ avoided	€/t_CO_2__	-	110.8	24.2	-	115.7	129.3

a The CO_2_ captured and the CO_2_ capture rate refer only to the additional CO_2_ capture sections of the reference and INITIATE plants. Therefore, the performance of the CO_2_ capture section present in conventional NH_3_ plants is not included.

In the reference steel mill scenario, the adoption of WGS+MDEA carbon capture technology leads to a reduction in internally produced power and to an increased consumption of natural gas for steam generation needed in solvent regeneration. This contrasts with INITIATE’s symbiotic approach, where ammonia synthesis efficiently utilizes byproduct gases like BOFG from steel production, leading to substantial natural gas savings. For the large-scale INITIATE plant, SPECCA is lower than that of the reference plant specifically when the carbon footprint of imported electricity is less than 255 kg_CO_2__/MWh. As already underlined, the large electricity consumption of the INITIATE large-scale plant is due to the absence of the power plant and the power required for compression of BFG and BOFG gas mixture to the operating pressure of WGS and SEWGS reactors. An important consideration is the high nitrogen content within BFG, which just in part contributes to ammonia synthesis. The excess nitrogen brings the drawback of increasing the overall power consumption of the INITIATE plant. Despite this, when analyzing scenarios with renewable energy sources, the SPECCA of the INITIATE large-scale plant is lower than that of reference plants, leading to negative values of the SPECCA indicator. The symbiotic system thus proves beneficial by facilitating primary energy savings compared with traditional setups where steel and chemical production processes operate independently, underscoring the potential for INITIATE plants to enhance sustainability through optimized resource utilization and reduced environmental impact.

The results of the economic assessment, obtained using the methodology exposed in Section 4, are detailed in Table 8. The breakdown of the levelized cost of hot rolled coil for the base, reference, and INITIATE steel mills, considering natural gas and electricity prices equal to 50 €/MWh and 125 €/MWh respectively, is shown. In the case of INITIATE, to permit a comparison among the cases, the revenues of the selling of urea are included considering a price equal to the levelized cost of urea computed for the base cases (Figure S1). The levelized cost of hot rolled coil increases from 528 €/t_HRC_ in the base case to 604 €/t_HRC_ in the reference case. For the small-scale INITIATE plant, the steel production cost slightly increases to 530 €/t_HRC_, whereas for the large-scale plant, it rises to 685 €/t_HRC_. “Other variable OPEX” includes the cost of coal, iron ores, scrap and ferroalloys, fluxes, consumables, miscellaneous OPEX of steel plant as well as of slag processing, disposal, and landfill. “Fixed O&M” includes the costs associated with maintenance as well as direct and indirect labor expenses. These costs remain nearly constant across the different cases, as the fixed operating expenditures of the steel plant constitute the predominant share of the total fixed O&M. “Other revenues” include the revenues from the selling of coke byproducts, slag and argon, whose selling price was taken from IEAGHG 2013 technical report on steel plants.^28^ Table S9 in the Supporting Information outlines the total plant cost for both small- and large-scale INITIATE plants. Notably, a significant portion of CAPEX is allocated to the steel plant infrastructure. A breakdown of annual expenses and revenues specific to the INITIATE plants is provided in Table S10 in the Supporting Information.

**Table 8:** Breakdown of the LCOHRC for the Base, Reference, and INITIATE Steel Mills Considering Natural Gas and Electricity Prices Equal to 50 €/MWh and 125 €/MWh

LCOHRC [€/t_HRC_]	Base	ref.	INITIATE Small-scale	INITIATE Large-scale
CAPEX	133.1	146.7	141.0	174.0
– Steel mill	133.1	146.7	132.6	126.6
– CO_2_ capture and NH_3_ production	0	0	6.2	35.7
– Urea production	0	0	2.2	11.7
OPEX	411.1	469.2	418.0	604.5
– Electricity	0	12.3	0	117.8
– Natural gas	0	9.8	2.5	19.3
– CO_2_ transport and storage	0	31.8	4.0	55.2
– Other variable OPEX	306.9	309.6	306.7	305.7
– Fixed OPEX	104.2	105.7	104.6	104.5
REVENUES	16.2	11.6	28.5	91.0
– Electricity	4.6	0	0	0
– Other revenues	11.6	11.6	11.6	11.6
– Urea	0	0	16.9	79.4
Total	528.0	604.4	530.3	685.5

#### 5.3.1 Sensitivity Analysis on Natural Gas and Electricity Prices

Since two different products, steel and urea, are produced in the base, reference, and INITIATE plants, a consistent comparison between the plant configurations was achieved by calculating the levelized cost of hot rolled coil while keeping the levelized cost of urea in the reference and INITIATE plants equal to the base case one (Figure S1). This approach enables a meaningful comparison among the different solutions despite steel and urea being produced in separate facilities in the base and reference cases. It is important to note that, therefore, all costs associated with the implementation of carbon capture and storage technologies are allocated to steel production. A sensitivity analysis was performed by varying the natural gas and electricity prices. The results are presented in Figure 8 and in Figure 9. In the base steel plant, which does not import natural gas, the levelized cost of the hot rolled coil is independent of the natural gas price, reflected by a single black dotted line (Figure 8). For small-scale plants, across all considered natural gas prices, the INITIATE plant consistently exhibits a lower steel production cost than the reference case. This is visually indicated by the continuous line remaining below the corresponding dotted line of the same color. In contrast, for large-scale plants, the INITIATE configuration achieves a lower LCOHRC primarily under scenarios of high natural gas prices. When natural gas prices decrease, INITIATE plants may still maintain a lower levelized cost of hot rolled coil, but this advantage is contingent on concurrent reductions in electricity prices. Similar trends are observed in Figure 9, which depicts the cost of CO_2_ avoided. This behavior results from increased electricity consumption and decreased natural gas import in the large-scale INITIATE plant.

**Figure 8:**
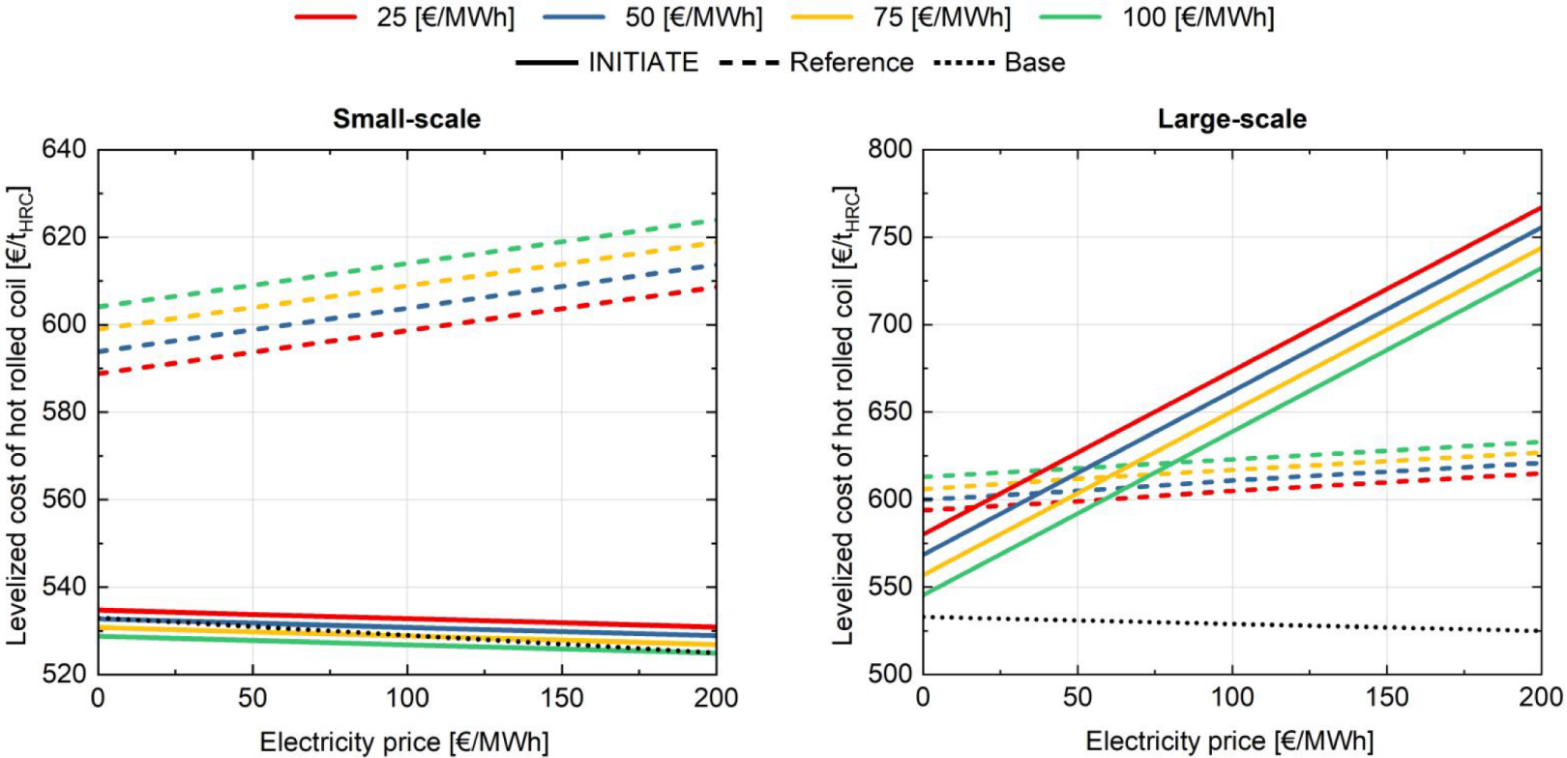
Comparison between the LCOHRC for the small- and large-scale INITIATE, reference, and base plants. Each color represents a different value of the natural gas price.

**Figure 9:**
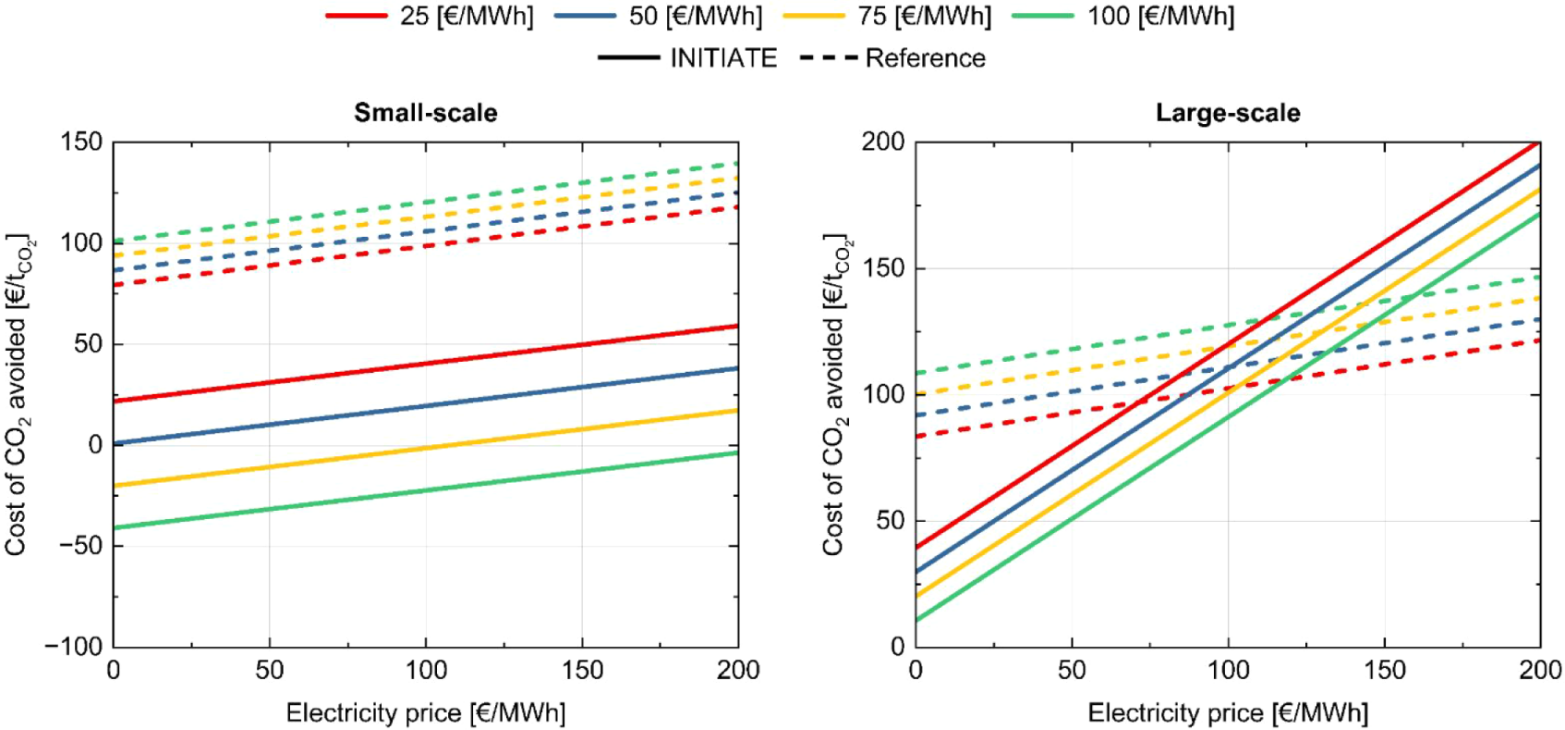
Cost of CO_2_ avoided for reference and INITIATE plants as a function of electricity and natural gas prices, considering a CO_2_ footprint of imported electricity equal to 250 kg_CO_2__/MWh. Each color represents a different value of the natural gas price.

#### 5.3.2 Cost of CO_2_ Avoided for Reference and INITIATE Plants in Europe

The average natural gas and electricity prices for 2024, along with the average CO_2_ footprint of the electricity grid for each European country shown in Figure 10, were used to calculate the variation in the cost of CO_2_ avoided (CCA). Price data for these commodities were sourced from Eurostat database.^53^ For Poland and North Macedonia, the average natural gas prices from the first half of 2024 were used due to the unavailability of data regarding the second half of the year. Similarly, CO_2_ footprint data for the electricity grid were primarily obtained from Eurostat database,^53^ except for Bosnia and Herzegovina, Moldova, North Macedonia, Serbia, and Turkey, where data were sourced from the Web site “electricitymaps.com”.^62^ Notably, in the reference cases, the range between the highest and lowest CCA values is narrower compared with the INITIATE plants, with this trend being especially pronounced in the large-scale configurations. Finland and Sweden present favorable conditions for the INITIATE plants thanks to the combination of low electricity prices, high natural gas prices, and low CO_2_ footprint of the electricity mix. In the case of large-scale INITIATE plants, which do not always achieve a lower cost of CO_2_ avoided than the reference configuration, the availability of low-cost electricity is a critical factor. Electricity imports in the large-scale INITIATE plant are substantially higher than in the other cases, making cheap electricity essential. As shown in Figure 9, at high electricity prices, the CCA of the large-scale INITIATE plant exceeds that of the corresponding reference plant, even under high natural gas prices. Although the CO_2_ intensity of electricity generation also affects the CCA, regions with low electricity costs typically coincide with low CO_2_ footprint of electricity from the grid. As illustrated in Figure 9, under low electricity price conditions, the large-scale INITIATE plant achieves a CCA lower than that of the reference case, even when accounting for a grid electricity footprint of 250 kg_CO_2__/MWh. In a low-emission electricity scenario, the advantage of INITIATE over the reference configuration becomes even more pronounced. For small-scale plants, however, the decisive factor is the natural gas price. Unlike the large-scale configuration, the small-scale INITIATE plant does not import electricity from the grid. Although some natural gas is imported, the amount remains lower than in the corresponding reference case. The largest difference in CCA between the small-scale INITIATE and the reference plant occurs at a natural gas price of 100 €/MWh; this difference narrows as natural gas prices decrease (Figure 9). Therefore, optimal deployment requires prioritizing regions with low electricity prices for large-scale INITIATE plants and regions with high natural gas prices for small-scale INITIATE plants.

**Figure 10:**
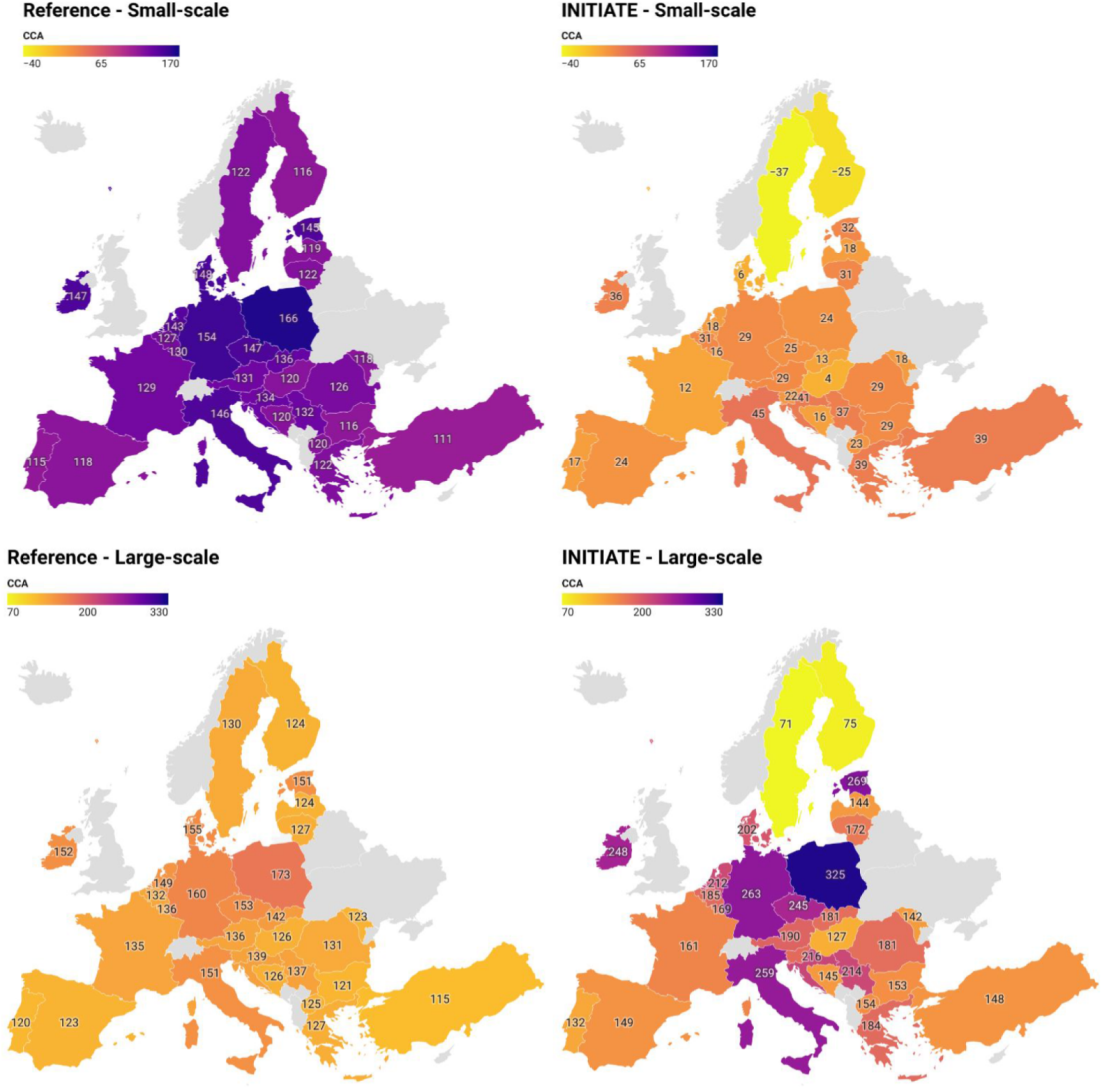
CCA [€/t_CO_2__] across European countries computed considering average values of natural gas and electricity prices and the CO_2_ footprint of the electricity mix in 2024 per each country.

## 6 Conclusions

The world is currently facing with the imperative to transition toward a net-zero society to mitigate the severe impacts of global warming. Iron and steel industry responsible of around 7% of global CO_2_ emissions is considered a hard-to-abate sector because of its reliance on fossil fuels. Similarly, the fertilizer sector represents a significant share of industrial CO_2_ emissions, relying on the use of fossil fuels both for energy purposes and as raw materials, such as in the Haber–Bosch process for the synthesis of ammonia. The work presented in this paper explores the advantages brought by the industrial symbiosis between these two sectors. Gas streams, such as BOFG and BFG, normally produced and used as fuels in the different processes of a conventional BF–BOF steel mill can be decarbonized by the means of SEWGS carbon capture technology and used as feedstock for ammonia and urea synthesis. In the SEWGS columns, CO_2_ is separated from an H_2_–N_2_ mixture that is used for the synthesis of ammonia, which is then combined with part of the CO_2_ separated for the manufacturing of urea. Different plant configurations were compared, the so-called base, reference, and INITIATE plants. The base plants are represented by conventional steel and ammonia/urea plants, while the reference plants integrate conventional carbon capture technologies such as amine scrubbing. On the other hand, in the INITIATE plants, differently from the base and reference cases, the production of steel and fertilizers is coupled and takes place at the same site. In addition, two different sizes of the ammonia/urea plants were considered, namely 224 t_urea_/day (small-scale) and 1500 t_urea_/day (large-scale). The small-scale INITIATE plant permits achieving a limited CO_2_ avoidance, around 5%, with respect to the base case since only BOFG is decarbonized through SEWGS technology. On the other hand, in the case of large-scale INITIATE plants, a CO_2_ avoidance ranging between 55% and 68% can be achieved depending on the CO_2_ footprint of the electricity imported from the grid. By the point of view of energy efficiency, the industrial symbiosis brings the advantage of reducing the consumption of resources. Indeed, the SPECCA index for the INITIATE plants can achieve negative values, meaning that both CO_2_ emissions and energy consumption are reduced. Considering the economic KPIs, the small-scale INITIATE plant always exhibits a levelized cost of hot rolled coil and a cost of CO_2_ avoided lower than the respective reference case. On the other hand, in the case of the large-scale plants, this happens for low electricity prices and high natural gas prices. However, large-scale INITIATE plant reaches a higher level of carbon avoidance. A comprehensive comparison thus should also consider the deeper decarbonization of the reference steel plant since higher costs and complexity associated with processing larger quantities of CO_2_ arise. The techno-economic assessment pointed out the importance of the parallel decarbonization of the electricity generation sector and of the availability of cheap electricity. Nevertheless, this is what is expected for the future with an increase of the share of renewables in the electricity energy mix.

Future work can consider a further reduction of emission in the large-scale INITIATE plant by decarbonizing flue gases from the sinter plant, either by adding a dedicated postcombustion section or by replacing the sinter plant with a pelletization plant. As already underlined, also for the reference steel mill, a case attaining a higher degree of CO_2_ emissions abatement could be considered. A different size of the ammonia section in the large-scale INITIATE plant could be considered, allowing for the production of the maximum possible quantities of ammonia and, consequently, urea. Alternatively, an optimization algorithm can be employed to determine the optimal size of the ammonia section in the INITIATE concept from a techno-economic perspective, taking into account the costs of key commodities, such as electricity and natural gas, at specific locations. Furthermore, an additional symbiotic configuration integrating an alternative CO_2_ capture technology to SEWGS, such as MDEA-based absorption, could be modeled. Finally, a concept similar to that of INITIATE could potentially be extended to the synthesis of other chemicals.

## Supplementary Material



## Notes

Additional results of the techno-economic assessment, process flow diagrams of the analyzed plants, stream specifications of the INITIATE plants, additional details about modeling of the plants in Aspen Plus, and values used for the calculation of components’ activities in the kinetic model of the ammonia reaction synthesis (PDF)

## Nomenclature

**Acronyms**
ASU: Air Separation Unit
BF: Blast Furnace
BFG: Blast Furnace Gas
BOF: Basic Oxygen Furnace
BOFG: Basic Oxygen Furnace Gas
CCA: Cost of CO_2_ Avoided [€/t_CO_2__]
CCR: CO_2_ Capture Ratio [%]
CCU: Carbon Capture Utilization and Storage
COG: Coke Oven Gas
CP: Carbon Purity [%]
DRI: Direct Reduced Iron
EAF: Electric Arc Furnace
EPC: Engineering Procurement and Construction
FCF: Fixed Charge Factor
FTR: Fired Tubular Reformer
GHG: Greenhouse Gas
HP: High Pressure
HRC: Hot Rolled Coil
IC: Indirect Costs
KPI: Key Performance Indicator
LCOA: Levelized Cost of Ammonia [€/t_NH_3__]
LCOHRC: Levelized Cost of Hot Rolled Coil [€/t_HRC_]
LCOU: Levelized Cost of Urea [€/t_urea_]
LP: Low Pressure
MDEA: Methyldiethanolamine
MEA: Monoethanolamine
MP: Medium Pressure
PEC: Primary Energy Consumption
SEWGS: Sorption Enhanced Water Gas Shift
SMR: Steam Methane Reforming
SPECCA: Specific PEC per unit of CO_2_ Avoided [GJ/t_CO_2__]
TAC: Total Annualized Cost [M€/y]
TDPC: Total Direct Plant Cost [M€]
TEC: Total Equipment Cost [M€]
TIC: Total Indirect Cost [M€]
TPC: Total Plant Cost [M€]
TRL: Technology Readiness Level
WGS: Water Gas Shift

## Symbols

e_CO2_: Specific CO_2_ emissions [t_CO_2__/t_product_]
h_eq_: Equivalent hours [h/y]
ṁ: Mass flow rate [kg/s]
p: Pressure [bar]
Δp: Pressure drops [bar]
T: Temperature [°C]
ΔT: Temperature difference [°C]

## Subscripts

h: Hydraulic
is: Isentropic
m: Mechanical
p: Polytropic
th: Thermal

## Greek

η: Efficiency [-]
