# Supplementary material for: Techno-Economic Assessment of Industrial Symbiosis Between Steel and Urea Plants: The INITIATE Process
Source: Energy Fuels. 2025 Nov 12;39(46):22293–310. doi: 10.1021/acs.energyfuels.5c04026 (PMC12641467; doi:10.1021/acs.energyfuels.5c04026)
Supplement: Supplementary file 1 [file ef5c04026_si_001.pdf]

Supporting Information

Techno-economic assessment of industrial symbiosis between steel and urea plants: the INITIATE process

Nicola Zecca*[a], Leonie Lücking [b], H.A.J van Dijk[b] and Giampaolo Manzolini[a]

[a.] Politecnico di Milano, Dipartimento di Energia, via Lambruschini 4, 20126, Milano, Italy.
[b.] TNO, Westerduinweg 3, 1755 LE, Petten, the Netherlands.

Contents

- Table S1: Details of CO_2 emissions of the base and reference BF-BOF steel mills and steel section of small- and large-scale INITIATE plants, considering a CO_2 footprint of electricity equal to 250 kg_{CO2}/MWh.
- Table S2: Details of CO_2 emissions of the base and reference ammonia + urea plants and chemical section of small- and large-scale INITIATE plants, considering a CO_2 footprint of electricity equal to 250 kg_{CO2}/MWh.
- Table S3: Breakdown of the LCOHRC and annual costs/revenues for the base and reference BF-BOF plants.
- Table S4: Main results of ammonia plants coupled with urea plants.
- Table S5: Main streams specifications for the small-scale INITIATE plant.
- Table S6: Main streams specifications for the large-scale INITIATE plant.
- Table S7: Property methods and Aspen Plus components used in the process modelling.
- Table S8: Details of the combined cycle of the base and reference BF-BOF steel mills and small-scale INITIATE plant.
- Table S9: Total plant cost of INITIATE small- and large-scale plants.
- Table S10: Breakdown of annual costs/revenues for the small- and large-scale INITIATE plants.
- Table S11: Values for the calculation of activities.
- Figure S1: Levelized cost of urea as a function of the electricity and natural gas price. Each colour represents a different value of the natural gas price.
- Figure S2: Levelized cost of hot rolled coil (LCOHRC) of reference and INITIATE plants computed varying the electricity and natural gas price.
- Figure S3: Gas distribution within the base BF-BOF steel mill.
- Figure S4: Gas distribution within the reference BF-BOF steel mill.
- Figure S5: Layout of the of the base ammonia plants.
- Figure S6: Layout of the reference ammonia plants.
- Figure S7: Layout of the small-scale urea plant based on conventional total recycling process.
- Figure S8: Layout of the large-scale urea plant based on CO2 stripping process
- Figure S9: Small-scale INITIATE plant – gas distribution.
- Figure S10: Large-scale INITIATE plant – gas distribution.

Additional results of the techno-economic assessment

Table S1 and Table S2 show the CO_2 emissions of the steel and chemical plants analysed in this work, considering a CO_2 footprint of electricity equal to 250 kg_{CO2}/MWh.

Table S1: Details of CO_2 emissions of the base and reference BF-BOF steel mills and steel section of small- and large-scale INITIATE plants, considering a CO_2 footprint of electricity equal to 250 kg_{CO2}/MWh.

Emission [kg_{CO2}/t_{HRC}]	Base BF-BOF	Reference BF-BOF	Small-scale INITIATE	Large-scale INITIATE
Iron ore production	67.3	67.3	67.3	67.3
Direct emissions	2068.0	1314.6	1954.6	650.8
- Flared	66.8	66.8	66.8	66.8
- Coke plant	194.1	194.1	194.1	30.7
- Lime plant	71.5	71.5	71.5	71.5
- Sinter plant	287.8	287.8	287.8	287.8
- Hot metal production	2.5	2.5	2.5	2.5
- Hot stoves	413.5	413.5	413.5	4.9
- Desulphurization	5.3	5.3	5.3	5.3
- BOF	8.1	8.1	8.1	8.1
- Ladle metallurgy	0.8	0.8	0.8	0.8
- Continuous caster	0.8	0.8	0.8	0.8
- Hot rolling mill	57.3	57.3	57.3	57.3
- BFG power plant	835.6	0.0	835.6	0.0
- BOFG power plant	123.8	0.0	0.0	0.0
- Decarbonised stream	0.0	165.0	0.0	33.8
- Natural gas combustion	0.0	41.0	10.4	80.5
Indirect emissions	-27.8	1.4	0.0	100.2
- Steel plant	0.0	12.2	0.0	73.2
- CO_2 capture	0.0	11.0	0.0	37.5
- CO_2 compression for storage	-27.8	24.6	0.0	210.9
Total CO_2 emissions	2107.4	1406.5	2021.9	929.0

Table S2: Details of CO_2 emissions of the base and reference ammonia + urea plants and chemical section of small- and large-scale INITIATE plants, considering a CO_2 footprint of electricity equal to 250 kg_{CO2}/MWh.

Emission [kg_{CO2}/t_{urea}]	Small-scale			Large-scale		
	Base	Reference	INITIATE	Base	Reference	INITIATE
Direct emissions	323.6	94.9	135.5	319.7	93.9	16.8
- Ammonia plant	323.6	28.0	135.5	319.7	28.6	16.8
- Additional natural gas combustion	0	66.8	0	0	65.3	0
Indirect emissions	199.8	208.3	0	11.5	19.2	151.7
- Indirect	199.8	199.8	0	11.5	11.5	151.7
- CO_2 compression for storage	0	8.5	0	0	7.7	0
Total CO_2 emissions	523.4	303.1	135.5	331.2	113.1	168.5

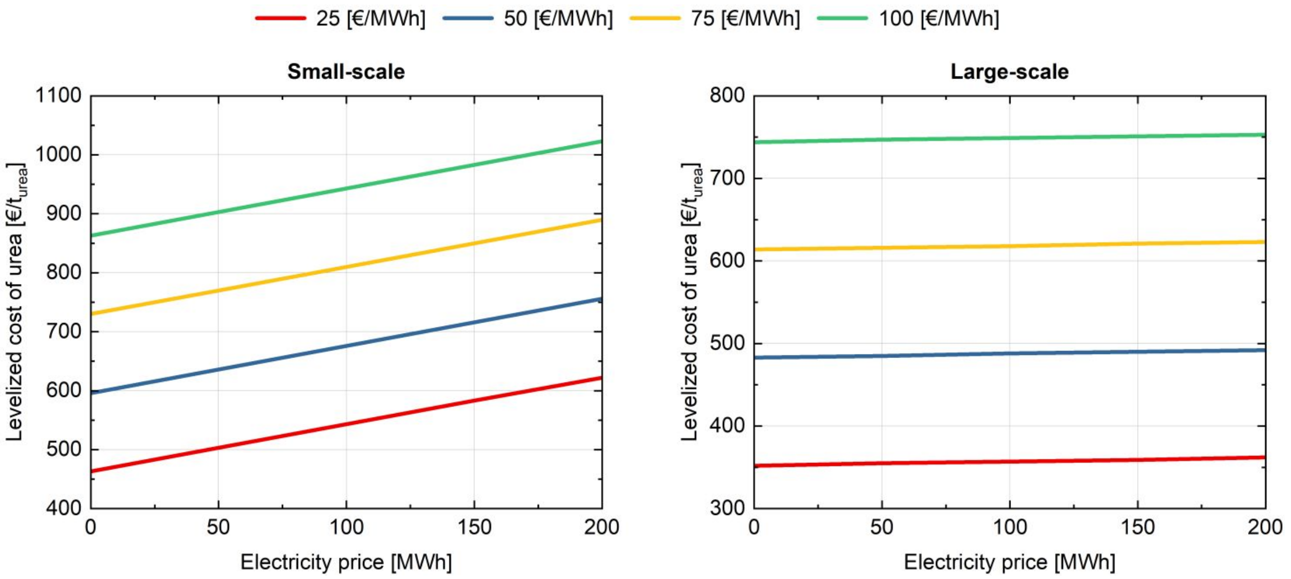

Figure S1: Levelized cost of urea as a function of the electricity and natural gas price. Each colour represents a different value of the natural gas price.

Figure S1 presents the levelized cost of urea (LCOU) for various combinations of natural gas and electricity prices, calculated for both the base small- and large-scale plant configurations. As emphasized in the article, this figure is kept constant across all plants to ensure consistency in the calculation of the levelized cost of steel and to enable a fair comparison. The LCOU is strongly influenced by the natural gas price, as natural gas serves as feedstock for ammonia production in the base cases. For small-scale ammonia and urea plants, where electricity is imported from the grid, the electricity price also plays a significant role in determining the overall production cost of urea. In contrast, for large-scale ammonia and urea plants, where most of the electricity demand is met by onsite generation, the influence of electricity price on the LCOU is comparatively limited.

The colour map in Figure S2 illustrates the levelized cost of hot rolled coil (LCOHRC), ranging from blue (indicating the lowest LCOHRC values) to red (indicating the highest LCOHRC values) for large-scale reference and INITIATE plants when varying electricity and natural gas prices. Results have been computed for five different European countries (Sweden, Italy, Germany, Spain, and Belgium) along with an average representation for the Eurozone, utilizing natural gas and electricity prices of 2024 from Eurostat [53]. As already underlined in the article, the small-scale INITIATE plant always shows a lower LCOHRC compared to the reference plant. On the other hand, in the case of large-scale plants, the INITIATE case shows a LCOHRC similar to the one of the reference case only in Sweden, while for the other countries the LCOHRC of the reference cases is lower. This is attributed to the combination of relatively low electricity prices and comparatively higher natural gas prices in Sweden, which optimize the cost-effectiveness of the INITIATE plant. Conversely, in other European countries represented in the analysis, the reference plants exhibit lower LCOHRC values. However, it is important to highlight the significant potential for higher carbon avoidance achievable by INITIATE large-scale plants. Unlike in reference steel plants where only flue gases from power plants are decarbonized, INITIATE large-scale plants permits to achieve a deeper CO_2 emissions reduction. It is also known that increasing the emission reduction quota in many cases leads to increased costs.

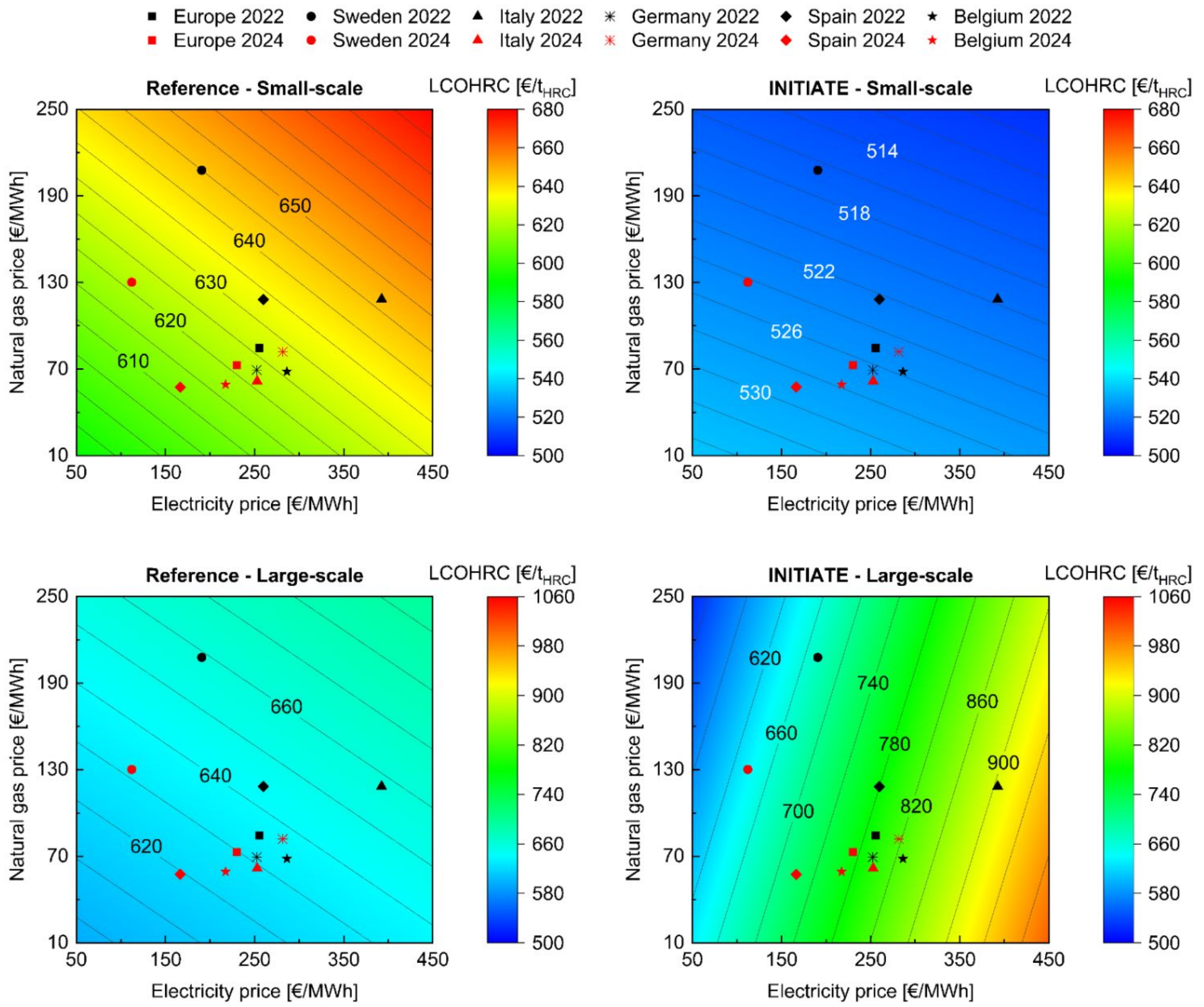

Figure S2: Levelized cost of hot rolled coil (LCOHRC) of reference and INITIATE plants computed varying the electricity and natural gas price.

Process flow diagrams of the analysed plants

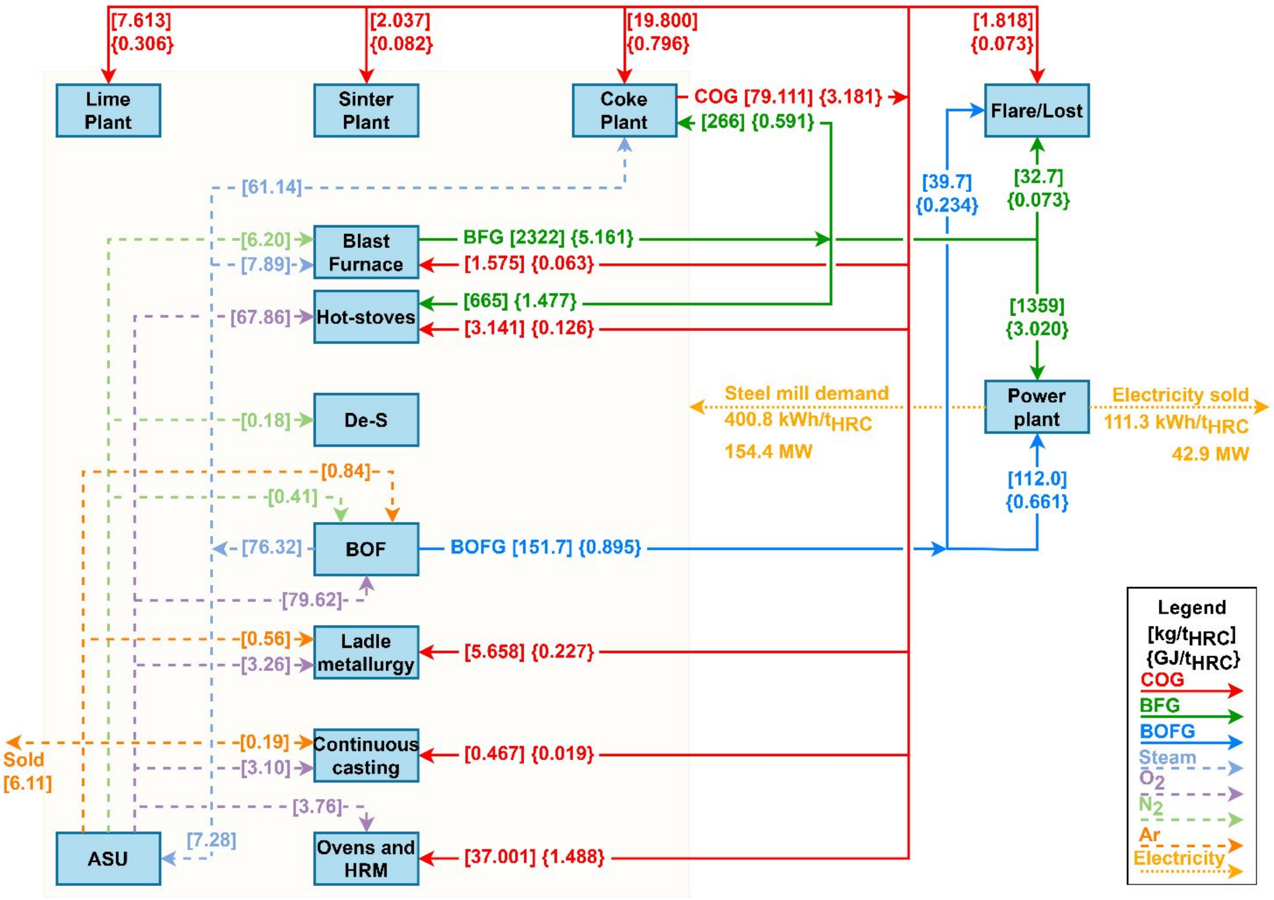

Figure S3: Gas distribution within the base BF-BOF steel mill.

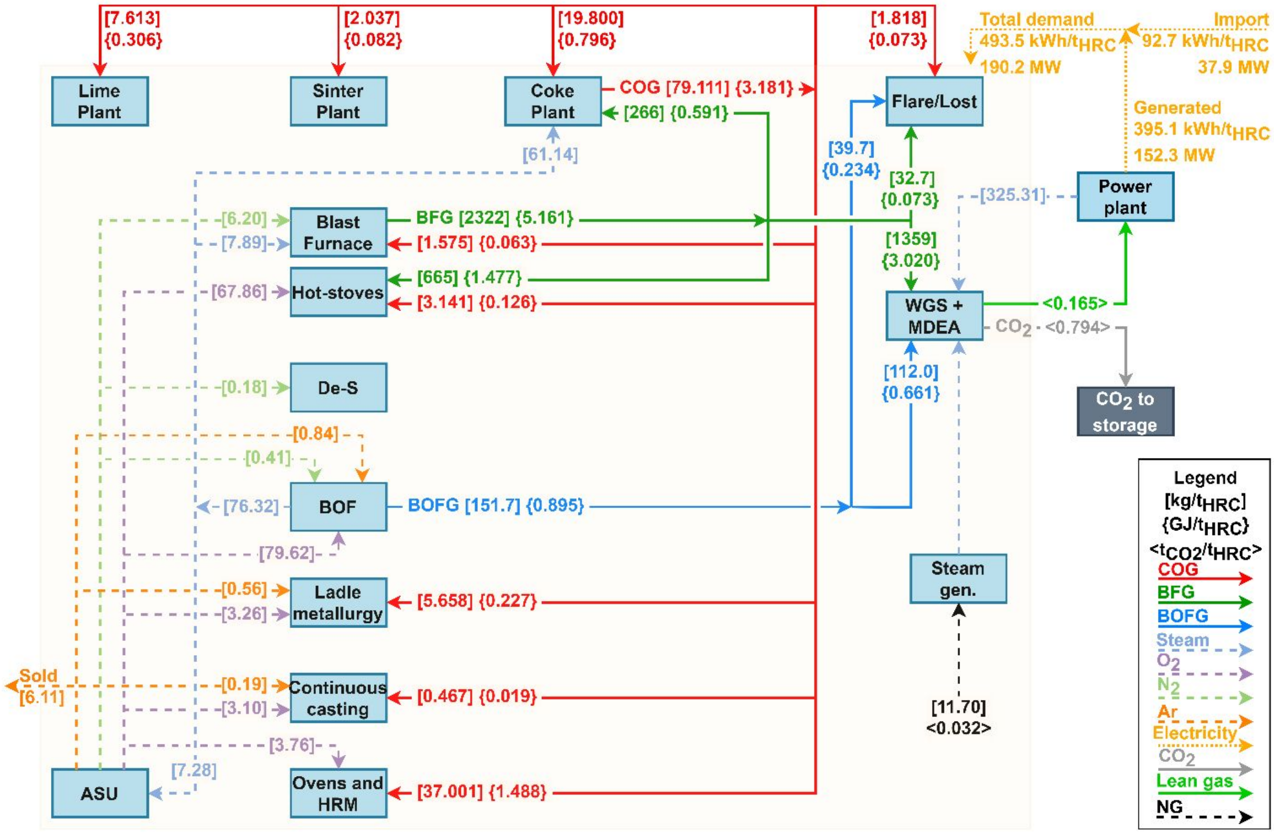

Figure S4: Gas distribution within the reference BF-BOF steel mill.

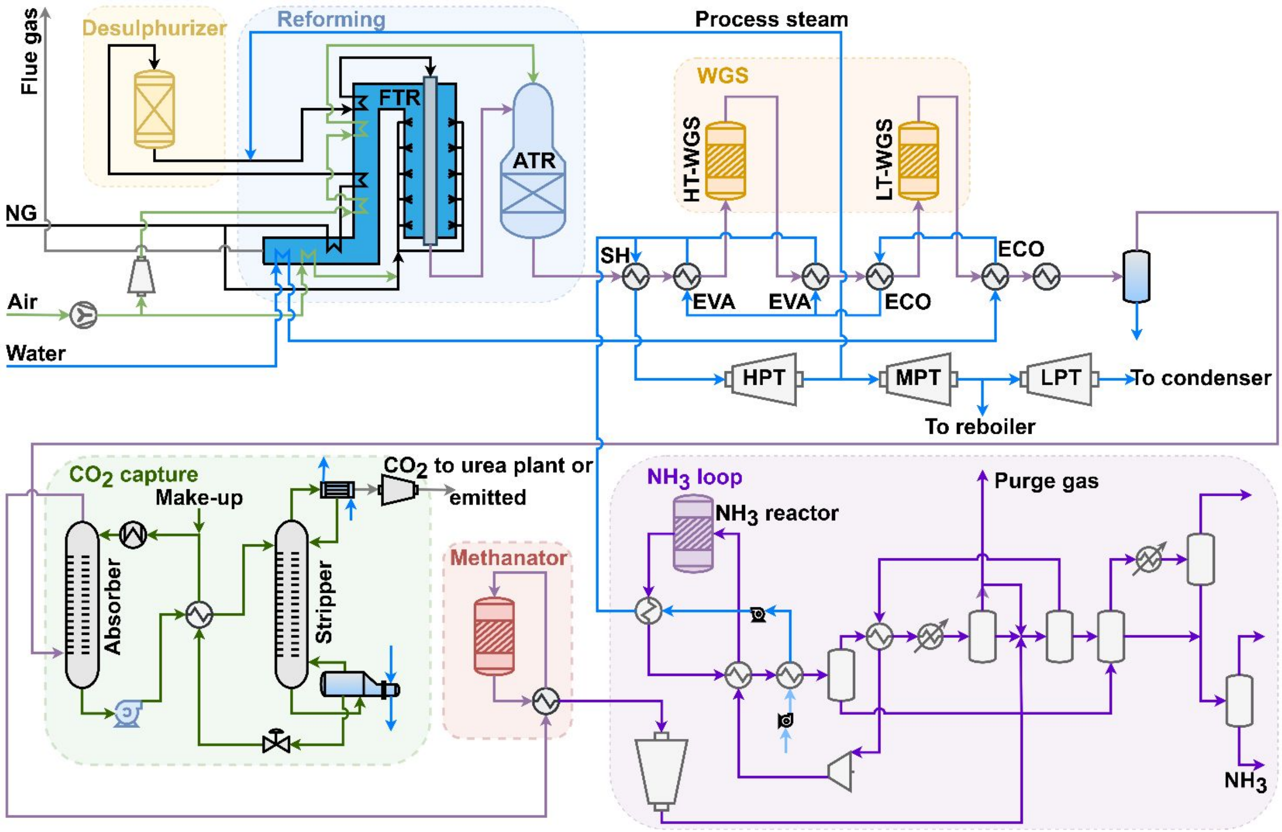

Figure S5: Layout of the of the base ammonia plants.

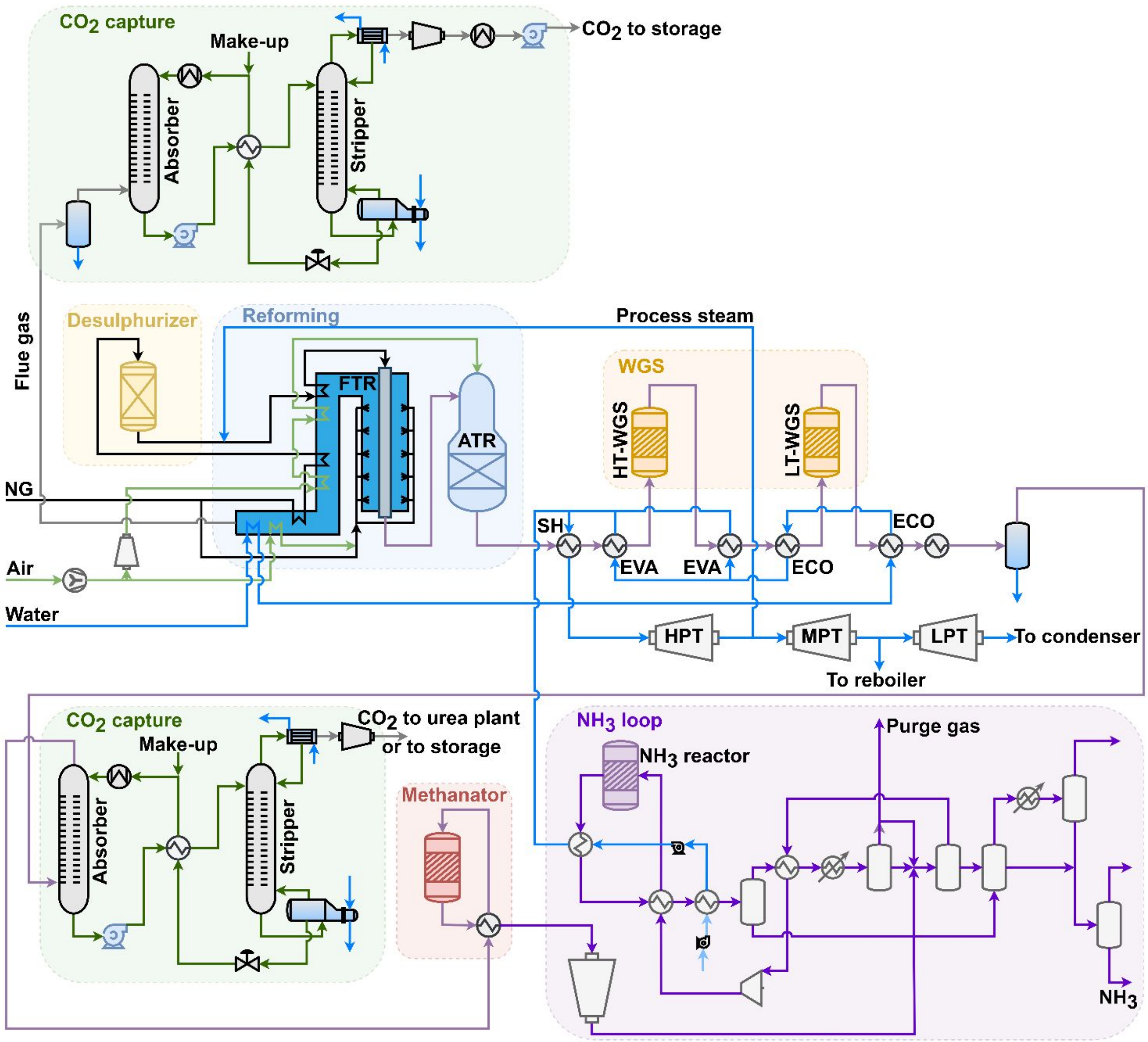

Figure S6: Layout of the reference ammonia plants.

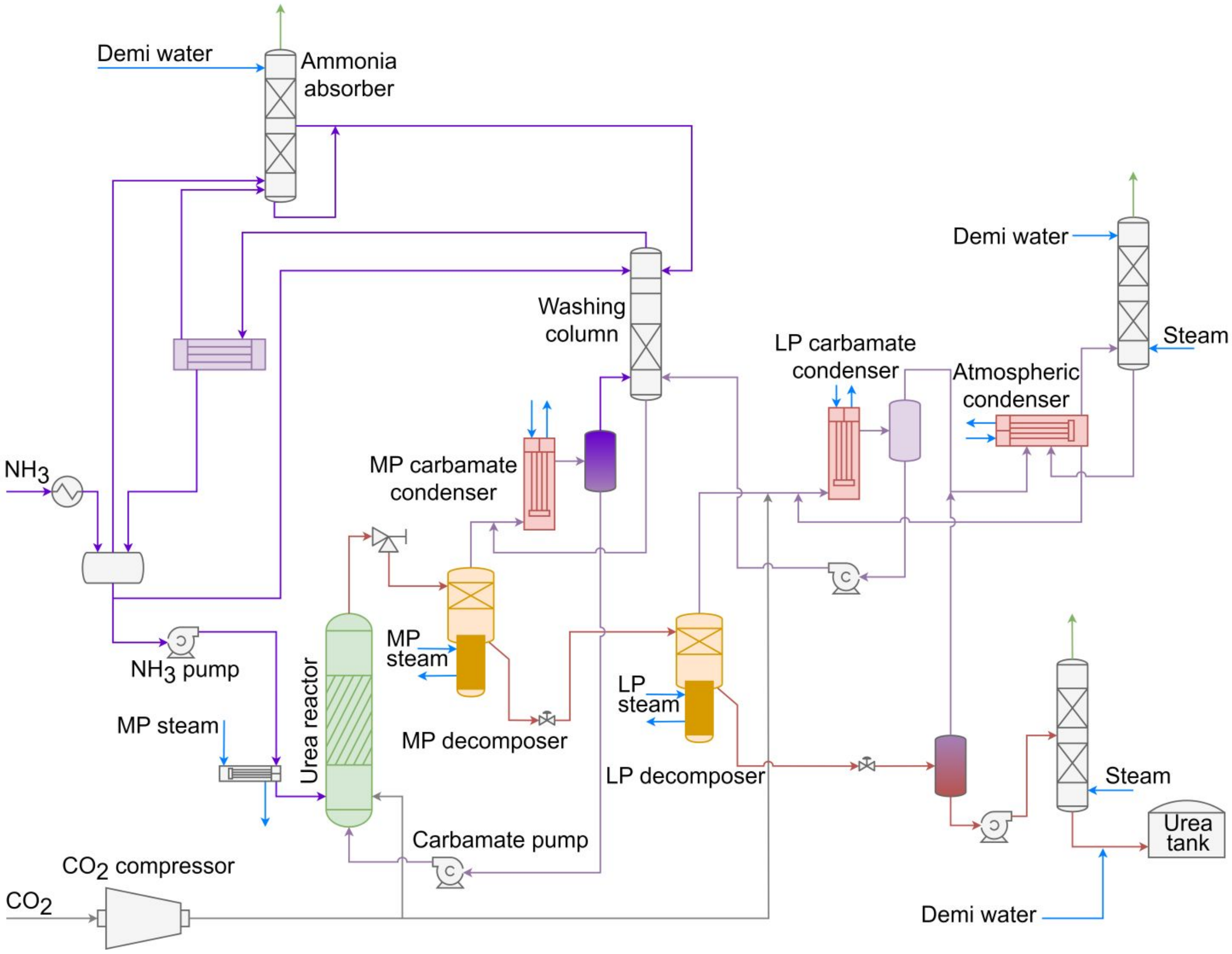

Figure S7: Layout of the small-scale urea plant based on conventional total recycling process.

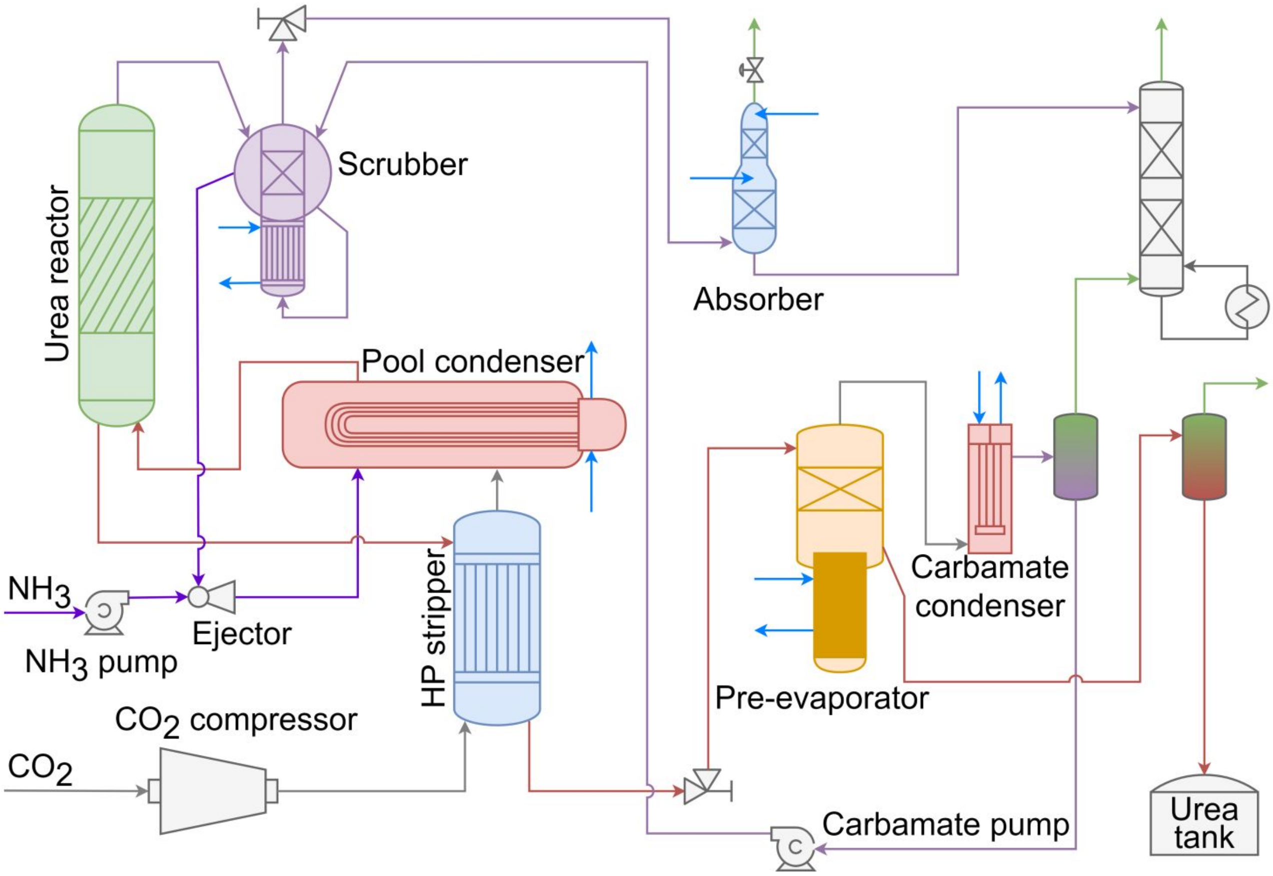

Figure S8: Layout of the large-scale urea plant based on CO_2 stripping process.

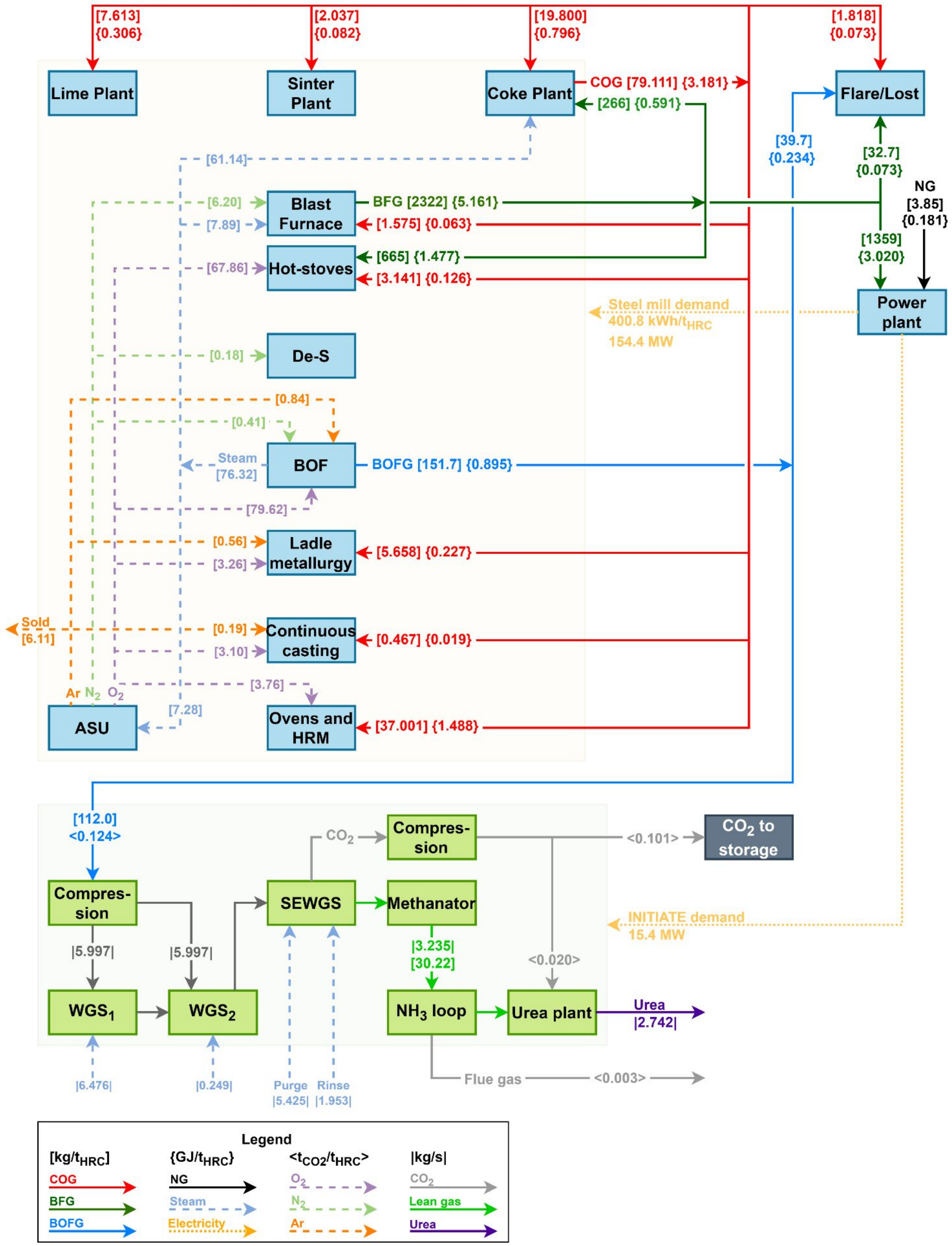

Figure S9: Small-scale INITIATE plant – gas distribution.

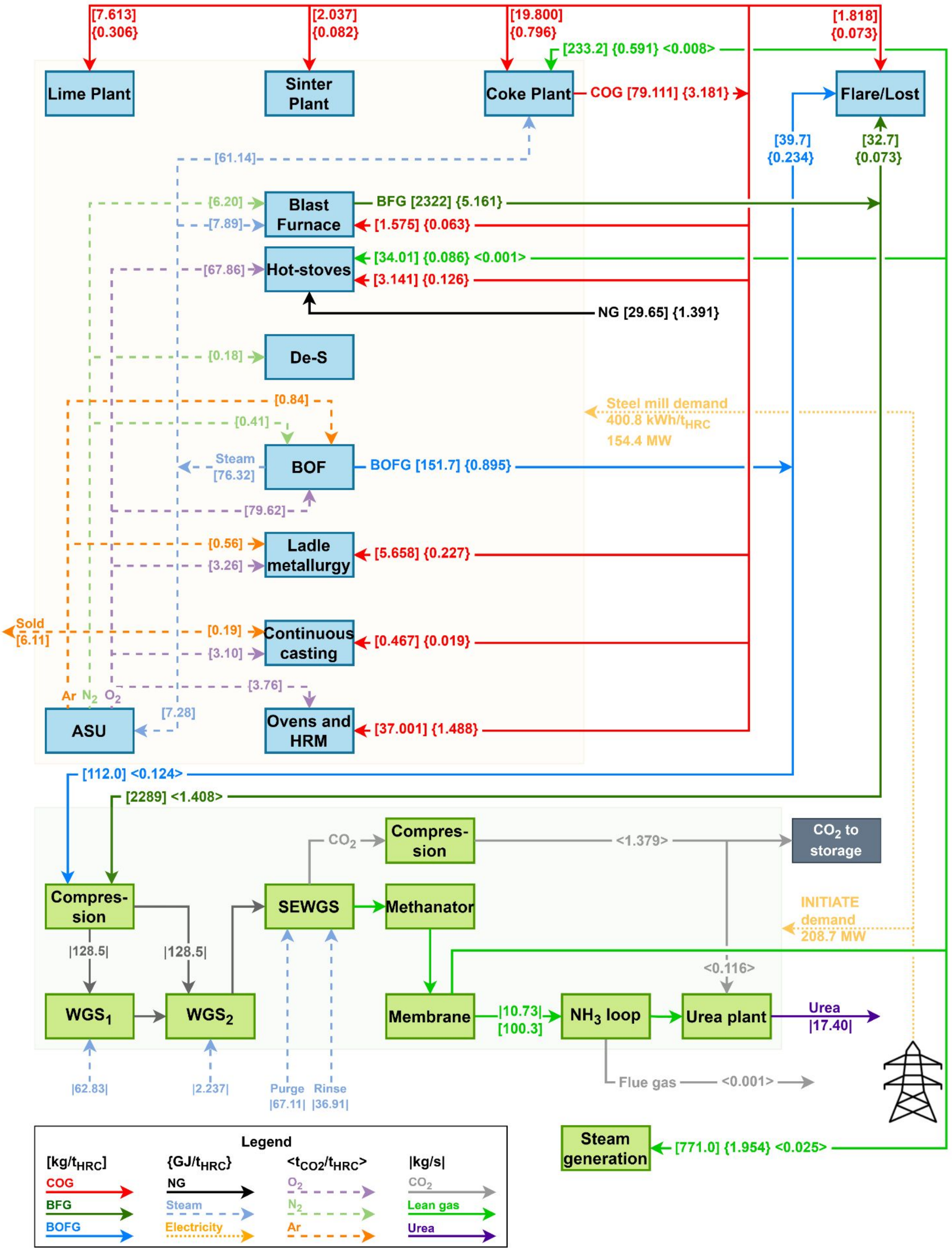

Figure S10: Large-scale INITIATE plant – gas distribution.

Economic results of base and reference BF-BOF steel plants

Table S3: Breakdown of the LCOHRC and annual costs/revenues for the base and reference BF-BOF plants.

Cost/Revenue	Base steel mill		Reference steel mill	
	**Annual cost [M€/y]**	**LCOHRC [€/t_{HRC}]**	**Annual cost [M€/y]**	**LCOHRC [€/t_{HRC}]**
**Annualised CAPEX**	**420.72**	**133.1**	**463.7**	**146.7**
**Fixed O&M costs**	**329.4**	**104.2**	**334.1**	**105.7**
- Maintenance	150.3	47.6	154.0	48.7
- Direct labour	130.6	41.3	131.5	41.6
- Indirect labour	48.6	15.4	48.6	15.4
**Variable O&M costs**	**904.1**	**286.1**	**1097.7**	**347.4**
- Fuel and reductant	331.22	104.8	362.3	114.6
- Iron ore (fines, lumps and pellets)	357.63	113.2	357.6	113.2
- Scrap and ferroalloys	159.54	50.5	159.5	50.5
- Fluxes	32.53	10.3	32.5	10.3
- Consumables & Other	37.78	12.0	46.4	14.7
- Electricity	-14.6	-4.6	38.9	12.3
- CO_2 transport and storage	-	-	100.4	31.8
**Miscellaneous OPEX costs**	**45.2**	**14.3**	**45.2**	**14.3**
- Miscellaneous work expense	36.6	11.6	36.6	11.6
- Other OPEX	8.6	2.7	8.6	2.7
**Other O&M costs**	**5.8**	**1.8**	**5.8**	**1.8**
- Slag processing	2.6	0.8	2.6	0.8
- Disposal and Landfill	3.2	1.0	3.2	1.0
**Revenues of coke by-products selling**	**15.6**	**4.9**	**15.6**	**4.9**
- Crude Tar	8.6	2.7	8.6	2.7
- Benzole	6.7	2.1	6.7	2.1
- Sulphur	0.3	0.1	0.3	0.1
**Revenues of slag selling**	**13.2**	**4.2**	**13.2**	**4.2**
- BF Slag	12.9	4.1	12.9	4.1
- BOS Slag	0.3	0.1	0.3	0.1
**Revenues of argon selling**	**7.8**	**2.5**	**7.8**	**2.5**
**Total**	**1668.60**	**528.0**	**1909.8**	**604.4**

Main results of ammonia plants coupled with urea plants

Table S4: Main results of ammonia plants coupled with urea plants.

Parameter	Unit	Small-scale plant		Large-scale plant	
		Base	Reference	Base	Reference
Capacity of urea plant	t_{urea}/day	224.21	224.21	1503.26	1503.26
Urea plant NH_3 input	t_{NH3}/day	128.25	128.25	849.34	849.34
Urea plant CO_2 input	t_{CO2}/day	173.84	173.84	1097.38	1097.38
Ammonia plant power consumption	MW_e	7.28	7.60	23.46	25.38
Urea plant power consumption	MW_e	1.40	1.40	12.25	12.25
Power imported from the grid	MW_e	7.46	7.78	2.88	4.81
Steam input (ammonia plant)	MW_{th}	-10.79	-8.03	-36.23	-18.06
Steam input (urea plant)	MW_{th}	10.79	10.79	36.17	36.17
Steam net input (ammonia + urea plants)	MW_{th}	0	2.76	-0.06	18.11
Total CO_2 emissions	t_{CO2}/day	117.35	67.97	497.85	169.99
Total CO_2 emissions saved	t_{CO2}/day	-	49.39	-	327.86
Total CO_2 to storage	t_{CO2}/day	-	66.29	-	437.62
Total direct plant cost CC section	M€	-	12.42	-	55.20
Total direct plant cost ammonia plant	M€	62.11	74.53	335.20	390.40
Total direct plant cost urea plant	M€	45.15	45.15	243.62	243.62
Total plant cost ammonia + urea plants	M€	173.76	193.88	937.69	1027.11
Total variable costs	M€/y	28.45	31.03	139.41	156.14
Total fixed costs	M€/y	8.60	10.12	23.76	26.71
Primary energy consumption	GJ/t_{urea}	22.66	23.93	19.01	20.24
Process carbon intensity	t_{CO2}/t_{urea}	0.52	0.30	0.33	0.11
CO_2 avoidance (CA)	%	-	42.08	-	65.86
SPECCA	GJ/t_{CO2}	-	5.78	-	5.67
Levelized cost of urea	€/t_{urea}	696	775	489	543
Cost of CO_2 avoided	€/t_{CO2}	-	357	-	250

Main streams specifications for the INITIATE plant

Table S5: Main streams specifications for the small-scale INITIATE plant.

Point	T	P	ṁ	Composition [%mol]							
	[°C]	[bar]	[kg/s]	H_2O	H_2	CO	CO_2	N_2	CH_4	NH_3	Ar
1	25.0	1.0	11.07	-	3.01	64.80	16.44	15.76	-	-	0.01
2	416.0	17.0	5.54	-	3.01	64.80	16.44	15.76	-	-	0.01
3	510.0	17.0	12.55	44.74	20.01	2.29	24.41	8.52	-	-	0.01
4	320.0	17.0	18.86	34.03	15.13	17.01	21.50	12.29	-	-	0.01
5	400.0	17.0	18.86	22.03	27.13	5.01	33.50	12.29	-	-	0.01
6	250.0	16.9	4.30	12.86	61.01	1.79	0.24	24.01	-	-	0.01
7	71.0	16.9	4.30	15.76	57.01	-	-	25.02	2.11	-	0.01
8	5.0	312.0	3.23	-	67.68	-	-	29.70	2.50	-	0.01
9	4.2	309.0	7.68	-	57.62	-	-	31.35	3.95	6.89	0.01
10	19.0	308.0	7.37	-	59.27	-	-	32.25	4.05	4.24	0.01
11	200.0	328.0	7.37	-	59.27	-	-	32.25	4.05	4.24	0.01
12	240.0	313.0	7.37	-	42.06	-	-	28.88	4.82	24.02	0.01
13	39.0	311.0	6.04	-	49.05	-	-	33.70	5.46	11.55	0.01
14	39.0	311.0	1.32	-	1.71	-	-	1.10	1.15	95.96	0.01
15	32.2	20.0	1.57	-	0.03	-	-	0.02	0.32	99.61	0.01
16	15.0	309.0	1.59	-	52.08	-	-	35.78	5.74	6.14	0.01
17	1.3	404.0	21.94	62.61	0.44	0.03	36.73	0.18	-	-	-
18	32.4	110.0	10.88	0.59	1.18	0.09	97.65	0.48	-	-	-
19	100.0	200.0	2.12	2.46	1.16	0.09	95.82	0.47	-	-	-
20	400.0	17.0	1.95	100	-	-	-	-	-	-	-
21	400.0	1.5	5.43	100	-	-	-	-	-	-	-

Table S6: Main streams specifications for the large-scale INITIATE plant.

**Point**	**T**	**P**	**ṁ**	**Composition [%mol]**							
	**[°C]**	**[bar]**	**[kg/s]**	**H_2O**	**H_2**	**CO**	**CO_2**	**N_2**	**CH_4**	**NH_3**	**Ar**
1	25.0	1.0	250.29	-	2.43	24.62	21.17	51.78	-	-	-
2	425.4	17.0	125.15	-	2.43	24.62	21.17	51.78	-	-	-
3	450.9	17.0	179.18	29.81	14.03	1.54	24.82	29.80	-	-	-
4	320.0	17.0	306.56	19.81	9.68	9.86	23.23	37.41	-	-	-
5	400.0	17.0	306.56	12.90	16.60	2.95	30.14	37.41	-	-	-
6	260.0	16.9	139.42	12.26	29.02	1.01	0.22	57.49	-	-	0.01
7	35.0	16.9	139.42	14.05	25.76	-	-	58.93	1.26	-	0.01
8	35.0	16.9	121.56	-	29.97	-	-	68.56	1.46	-	0.01
9	35.0	2.0	1.74	-	100	-	-	-	-	-	-
10	35.0	16.9	110.83	-	18.33	-	-	79.96	1.70	-	0.01
11	312.0	5.0	10.73	-	74.59	-	-	24.87	0.53	-	-
12	310.0	3.9	33.05	-	67.78	-	-	22.81	2.46	6.95	0.01
13	18.2	309.0	31.10	-	70.03	-	-	23.56	2.53	3.88	0.01
14	200.0	328.0	31.10	-	70.03	-	-	23.56	2.53	3.88	0.01
15	240.0	313.0	31.10	-	53.53	-	-	18.11	3.05	25.31	0.01
16	39.0	312.0	22.93	-	64.16	-	-	21.71	3.52	10.59	0.01
17	39.0	312.0	8.17	-	2.14	-	-	0.69	0.76	96.40	-
18	32.5	20.0	9.83	-	0.04	-	-	0.02	0.23	99.71	-
19	10.1	310.0	0.61	-	68.36	-	-	23.14	3.71	4.78	0.01
20	395	1.3	271.15	62.06	0.28	0.01	37.09	0.55	-	-	-
21	25.7	110.0	149.43	0.59	0.72	0.04	97.19	1.45	-	-	-
22	100.0	200.0	12.65	2.46	0.71	0.04	95.37	1.42	-	-	-
23	400.0	17.0	36.91	100	-	-	-	-	-	-	-
24	400.0	1.5	67.11	100	-	-	-	-	-	-	-
25	35.0	16.9	28.29	-	18.33	-	-	79.96	1.70	-	0.01

Property methods and Aspen Plus components used in the process modelling.

Table S7: Property methods and Aspen Plus components used in the process modelling.

Plant	Aspen Plus ID	Comments / Specifications
**Ammonia plant - (RKS-BM property method + ELECNRTL in the CO_2 removal section)**		
NG desulphurization	Sep2 + RStoic	$S + H_2 \rightleftharpoons H_2S$; P_{in} = 35 bar; T_{in} = 345 °C; T_{out} = 325 °C
Fired tubular reformer	RStoic + RPlug	RStoic for reformer furnace; RPlug for reforming reactions; P_{in} = 35 bar; T_{in} = 495 °C
Auto thermal reformer	RPlug	P_{in} = 31 bar; T_{in} = 900 °C
High temperature shift	REquil	$CO + H_2O \rightleftharpoons CO_2 + H_2$; pressure = 0; duty = 0; T_{in} = 380 °C; T_{out} = 440 °C
Low temperature shift	REquil	$CO + H_2O \rightleftharpoons CO_2 + H_2$; pressure = 0; duty = 0; T_{in} = 210 °C; T_{out} = 230 °C
CO_2 removal	2 RadFraq + HeatX + Pump	Further details in the dedicated sections
Methanation	RGibbs	Calculate phase equilibrium and chemical equilibrium; pressure = 0; duty = 0; T_{in} = 280 °C
Syngas compressor	MCompr	3 stages, $T_{intercooling}$ = 15 °C
NH_3 Synthesis	4 RPlug + Heater(s) + Compr	The reactions are defined in Fortran subroutine using RPlug based on kinetics of [17]; P_{in} = 292 bar; T_{in} = 180 °C; T_{out} = 445 °C
Cooling cycle	Compr + 2 Heater + Valve	
**Small-scale urea plant - (SR-POLAR property method)**		
Urea reactor	RPlug	The reactions are defined in Fortran subroutine using RPlug; NH_3/CO_2 ratio = 4; conversion of 68% of CO_2 input; P = 195 bar
MP, LP, atmospheric decomposer	RStoic + 2 Flash2 for MP and LP; Flash2 for atmospheric	$CARBAMATE \rightleftharpoons 2NH_3 + CO_2$ (CARBAMATE conversion specified: MP = 68%, LP = 100%); HP = 19.6 bar; MP = 4.12 bar; LP = 1.47 bar
MP, LP, atmospheric condenser	RStoic	$2NH_3 + CO_2 \rightleftharpoons CARBAMATE$; CO_2 conversion specified: MP = 90%, atmospheric = 50% or NH_3 conversion specified: LP = 85%
Absorber, washing and other columns	RadFraq	Equilibrium
CO_2 compressor	MCompr	4 stages, $T_{intercooling}$ = 28 °C
**Large-scale urea plant - (SR-POLAR property method)**		
Urea reactor	RPlug	The reactions are defined in Fortran subroutine using RPlug; NH_3/CO_2 ratio = 3 with CO_2 via CO_2 stripper; T = 180 °C; P = 138 bar
CO_2 stripper	RadFrac	Equilibrium; 10 stages; condenser: none; reboiler: none; P = 138 bar
Pool condenser	RStoic	T = 167 °C; P = 138 bar; NH_3 conversion = 40.2%
Scrubber	RadFrac	Equilibrium; 5 stages; condenser: none; reboiler: Kettle; reboiler duty = -5.46 MW; P = 138 bar
Pre-evaporator	RStoic	$CARBAMATE \rightleftharpoons 2NH_3 + CO_2$; CARBAMATE conversion = 100%
Carbamate condenser	RGibbs	Calculate phase equilibrium and chemical equilibrium; Pressure = 138 bar; Temperature = 72.4 °C
**INITIATE plants - (RKS-BM property method)**		
WGS reactors	REquil	$CO + H_2O \rightleftharpoons CO_2 + H_2$; pressure = 0; duty = 0; T_{in} = 320 °C; P_{in} = 17 bar
SEWGS	RStoic + Sep + 2 Heater	$CO + H_2O \rightleftharpoons CO_2 + H_2$; pressure = 0; duty = 0; T_{in} = 400 °C; a dedicated calculator computes the CO conversion and composition and mass flow rate of outlet streams; CCR = 97.5%; rinse steam: T = 400 °C, P = 17 bar, H_2O/C ratio = 0.36 mol_{H2O}/mol_C for the small-scale plant and H_2O/C ratio = 0.55 mol_{H2O}/mol_C for the large-scale plant; purge steam: T = 400 °C, P = 1.45 bar, H_2O/C ratio = 1 for both small- and large-scale plants
Methanator	RGibbs	Calculate phase equilibrium and chemical equilibrium; pressure = 0; duty = 0; T_{in} = 250 °C
Membrane	Separator + Mixer + FSplit	A dedicated calculator computes the mass flow rate and composition of permeate and retentate streams
Syngas compressor	MCompr	3 stages; P_{out} = 312 bar; $T_{intercooling}$ = 15 °C
NH_3 loop compressor	Compr	P_{out} = 325 bar
NH_3 Synthesis	RPlug + Heater(s) + Compr	The reactions are defined in Fortran subroutine using RPlug based on kinetics of [17]; P_{in} = 325 bar; T_{in} = 200 °C; T_{out} = 500 °C

**MEA / MDEA CC capture sections - (ELECNRTL / ENRTL-RK property method)**		
HT WGS (only MDEA case)	RGibbs	$CO + H_2O \rightleftharpoons CO_2 + H_2$<br>Pressure = 0; Duty = 0;
Absorber	RadFraq	Equilibrium; 20 stages; condenser: none; reboiler: none
Stripper	RadFraq	Equilibrium; 20 stages; condenser: partial-vapor-liquid; reboiler: Kettle
Regenerative heat exchanger	HeatX	Pinch point ΔT = 10°C
Pump	Pump	$P_{out, MEA}$ = 3 bar; $P_{out, MDEA}$ = 6 bar η_h = 0.75; η_m = 0.95
Expander (only MDEA case)	Compr	Discharge pressure = 110 bar; η_{is} = 0.85; η_m = 0.95
**CO_2 compression train - (RKS-BM property method)**		
CO_2 compressor	MCompr	3 stages; P_{out} = 80 bar;<br>$\eta_{p,1,2}$ = 0.8; $\eta_{p,3}$ = 0.75; η_m = 0.95; $T_{intercooling}$ = 28 °C; $\Delta P_{intercooler,1}$ = 0.05 bar; $\Delta P_{intercooler,2}$ = 0.19 bar
CO_2 pump	Pump	Discharge pressure = 110 bar; η_h = 0.75; η_m = 0.95
**Waste heat recovery in plants - (STEAMNBS property method)**		
Economiser, evaporator superheater	HeatX	

Results of the INITIATE plants

Table S8: Details of the combined cycle of the base and reference BF-BOF steel mills and small-scale INITIATE plant.

Parameter	Unit	Base BF-BOF	Reference BF-BOF	Small INITIATE
Gas turbine power	MW	284.19	288.61	244.36
HP steam turbine power	MW	16.52	12.50	14.33
MP steam turbine power	MW	18.66	14.68	16.17
LP steam turbine power	MW	31.50	-	27.26
Air compressor power	MW	-97.82	-112.33	-79.35
Fuel compressor power	MW	-49.61	-45.81	-47.57
HP pump power	MW	-0.65	-0.53	-0.56
IPP pump power	MW	-0.17	-0.14	-0.15
LP pump power	MW	-0.03	-	-0.03
Capture plant power demand	MW	-	-35.71	-
Net power	MW	197.32	152.25	169.93
Thermal energy input	MW	394.05	368.77	342.63
Combined cycle Efficiency	%	50.07	41.29	49.60
Plant power requirement	MW	154.45	190.16	169.85
Electricity purchased	MW	-42.87	37.91	0
TEC combined cycle	M€	175.95	135.32	161.74

Table S9: Total plant cost of INITIATE small- and large-scale plants.

Equipment	Small-scale		Large-scale	
	TPC [M€]	Fraction [%]	TPC [M€]	Fraction [%]
Compressors	30.1	0.6	173.5	3.0
CO_2 compressor to storage	31.1	0.7	179.8	3.1
Pumps	3.5	0.1	4.1	0.1
Heat exchangers	92.8	2.0	326.5	5.6
Combustors	17.4	0.4	108.5	1.8
Water gas shifts	5.3	0.1	22.8	0.4
Methanator	2.2	0.0	9.4	0.2
Ammonia reactor	7.3	0.2	25.0	0.4
SEWGS	18.8	0.4	267.4	4.6
Membrane	0.0	-	87.4	1.5
Steel plant	4473.4	94.1	4271.9	72.8
Urea plant	73.1	1.5	394.7	6.7
Total	4755.0	100	5871.0	100

Table S10: Breakdown of annual costs/revenues for the small- and large-scale INITIATE plants.

**Cost/Revenue**	**Annual cost [M€/y]**	
	**Small-scale**	**Large scale**
**Annualised CAPEX steel plant**	**419.1**	**400.2**
**Annualised CAPEX chemical section**	**26.4**	**149.8**
**Fixed O&M costs**	**329.1**	**322.6**
- Maintenance	149.9	145.9
- Direct labour	130.6	128.2
- Indirect labour (steel mill)	48.6	48.6
- Fixed O&M chemical section	1.3	7.5
**Variable O&M costs**	**938.4**	**1518.9**
- Natural gas	7.9	61.0
- Fuel and reductant	331.2	331.2
- Iron ore (fines, lumps and pellets)	357.6	357.6
- Scrap and ferroalloys	159.5	159.5
- Fluxes	32.5	32.5
- Consumables and Other	36.8	30.5
- Electricity steel plant	0	158.3
- Electricity chemical section	0	213.9
- CO_2 transport and storage	12.8	174.3
**Miscellaneous OPEX costs**	**46.4**	**55.4**
- Miscellaneous work expense	36.6	36.6
- Other OPEX steel plant	8.6	8.6
- Other OPEX chemical section	0.6	3.7
**Other O&M cost steel plant**	**5.8**	**5.8**
- Slag processing	2.6	2.6
- Disposal and Landfill	3.2	3.2
**Revenues of coke by-products selling**	**15.6**	**15.6**
- Crude Tar	8.6	8.6
- Benzole	6.7	6.7
- Sulphur	0.3	0.3
**Revenues of slag selling**	**13.3**	**13.3**
- BF Slag	12.9	12.9
- BOS Slag	0.3	0.3
**Revenues of argon selling**	**7.8**	**7.8**
**Total**	**1728**	**2410**

Values used for the calculation of activities in the kinetic model of the ammonia reaction synthesis

Table S11: Values for the calculation of activities.

**i**	A_i	B_i	C_i
H_2	0.1975	0.02096	$5.04 \cdot 10^{2}$
N_2	1.3445	0.05046	$4.20 \cdot 10^{4}$
NH_3	2.393	0.03415	$4.77 \cdot 10^{6}$
Ar	1.2907	0	0
CH_4	2.2769	0	0
